# DNA Origami “Quick” Refolding inside of a Micron-Sized Compartment

**DOI:** 10.3390/molecules25010008

**Published:** 2019-12-18

**Authors:** Taiki Watanabe, Yusuke Sato, Hayato Otaka, Ibuki Kawamata, Satoshi Murata, Shin-Ichiro M. Nomura

**Affiliations:** 1 Department of Robotics, Graduate School of Engineering, Tohoku University, Sendai 980-8579, Japan; watanabe@molbot.mech.tohoku.ac.jp (T.W.); sato.y.cf@m.titech.ac.jp (Y.S.); otaka@molbot.mech.tohoku.ac.jp (H.O.); kawamata@molbot.mech.tohoku.ac.jp (I.K.); murata@molbot.mech.tohoku.ac.jp (S.M.); 2 Department of Computer Science, Tokyo Institute of Technology, Kanagawa 226-8502, Japan

**Keywords:** DNA origami, microcompartment, refolding

## Abstract

Investigations into the refolding of DNA origami leads to the creation of reconstructable nanostructures and deepens our understanding of the sustainability of life. Here, we report the refolding of the DNA origami structure inside a micron-sized compartment. In our experiments, conventional DNA origami and truss-type DNA origami were annealed and purified to remove the excess staples in a test tube. The DNA origami was then encapsulated inside of a micron-sized compartment of water-in-oil droplets, composed of neutral surfactants. The re-annealing process was then performed to initiate refolding in the compartment. The resulting 100-nm-sized DNA nanostructures were observed using atomic force microscopy (AFM), and the qualities of their structures were evaluated based on their shape. We found that the refolding of the DNA origami structure was favored inside the droplets compared with refolding in bulk solution. The refolded structures were able to fold even under “quick” one-minute annealing conditions. In addition, the smaller droplets (average diameter: 1.2 µm) appeared to be more advantageous for the refolding of the origamis than larger droplets. These results are expected to contribute to understanding the principles of life phenomena based on multimolecular polymer self-assembly in a micron-sized compartment, and for the production and maintenance of artificially designed molecules.

## 1. Introduction

All living organisms maintain life through the synthesis and function of a vast number of biopolymers. Most elongated polymers function in their folded states; however, polymers can often become unfolded—and thus dysfunctional—due to environmental changes, such as pH, salt concentration, hydrophobic balance, and cell cycle. Therefore, the living cell inevitably employs a refolding mechanism to recover from the unfolded and/or misfolded state of the polymer. For example, the self-refolding of proteins [1], molecular chaperones [2] that refold denatured or misfolded proteins, and chromatin re-modelling complexes [3,4] that reproduce the chromatin structure of genomic DNA, have been identified. In the field of nanoengineering, the folding of artificially designed DNA has been a rapidly emerging field [5,6,7,8]. DNA origami is one of the major structures in DNA nanotechnology. The DNA origami is formed by the cooperative folding of one long, single-stranded DNA (ssDNA) “scaffold” and hundreds of various short ssDNA “staples”, through a controlled temperature-changing processes called annealing [9,10]. The folding of DNA origami is a multimolecular self-assembly process and is more complicated than single-molecule folding. Commonly, a buffer solution in which scaffolds and excess staples (five to ten-fold larger amounts than scaffold) is annealed to obtain a DNA origami structure [10]. In addition, the annealing process requires several hours, depending on the structural complexity of the DNA origami. A purification to remove excess staples should be utilized before using the designed DNA structure. For using the purified DNA origami devices, the conditions should be carefully chosen to prevent their unfolding in low ionic strength solutions—such as physiological fluids or organic solvents—and kept at temperatures above 50 °C [11]. Once the DNA origami becomes unfolded, refolding is difficult because the staples become diluted in the solution and move away from the template during the annealing process. When the purified DNA origami exists in a closed space, is refolding possible? Here, we considered a micron-sized compartment within the solution, that was able to encapsulate the biopolymer. Micro-compartments can be used by live cells as reaction fields for encapsulated molecules and have unique properties, such as negligible levels of polymer exchange with the external environment and a high surface area to volume ratio, and diffusion does not limit reaction rates in these compartments [12,13]. In fact, in isolated microcompartments, such as water-in-oil droplets or liposomes, several reports have shown that molecular reactions in limited volumes demonstrate unique behaviors compared with those in bulk solution systems; for instance, increases in their reaction rates may occur, such as the chemical synthesis of imines [14], de novo protein synthesis reactions [13,15,16], and protein beta-sheet formation [17]. Therefore, in this study, we investigated the refolding efficiency of 100-nm-sized DNA origami structures, by annealing within a finite space of several micrometers in diameter.

## 2. Results

In this study, two types of planar DNA origami were designed, conventional DNA origami with normal crossover branching [9] and truss DNA origami [18,19], and their structures were confirmed by imaging using atomic force microscopy (AFM). First, in bulk solution, the mixtures of the scaffold and staple ssDNAs were thermally annealed, from 70 °C to 25 °C, over a 7.5 h period (5 min at 70 °C, followed by cooling to 25 °C, with a cooling rate of −0.1 °C/min).

**Figure 1:**
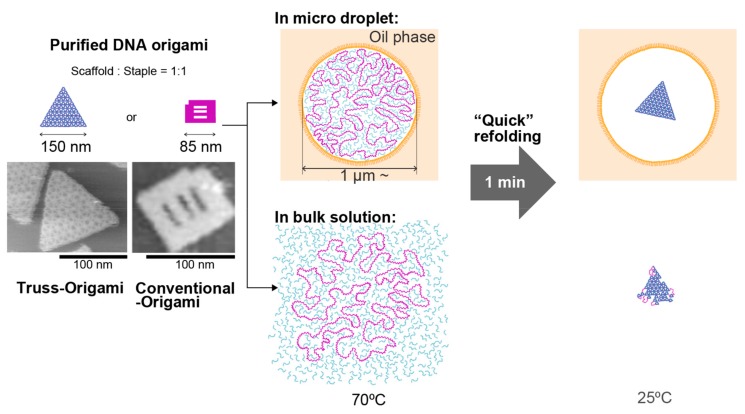
Schematic diagram of the re-folding experiment for the DNA origami structure. The DNA origami was assembled by the normal annealing process, purified, and used for refolding experiments after the removal of excess staples. The refolding process was performed in bulk solution and in the microcompartment of a water-in-oil droplet.

**Figure 2:**
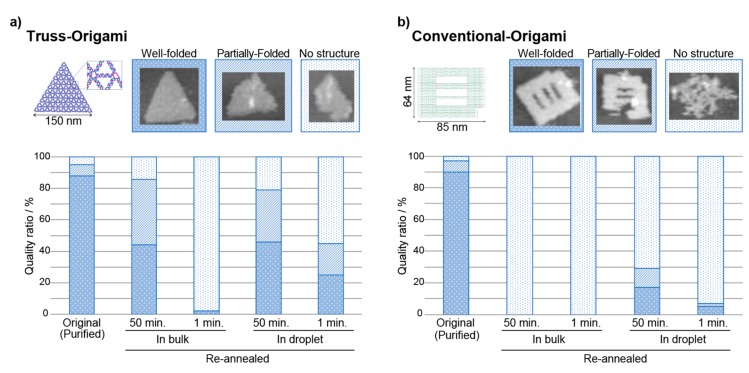
The refolding efficiencies of different DNA origami purified structures. The individual shapes of the structures were observed with atomic force microscopy (AFM), and the quality was judged according to three stages (well-folded, folded, and no structure). (**a**) truss origami. (**b**) The conventional origami structure. In bulk solution, the refolding efficiency of the truss structure was higher than that of the conventional origami. In the microcompartment droplet, over 20% of the refolded truss structure was observed, even under the “Quick_1min” annealing conditions.

The folded DNA origami structures were purified using polyethylene glycol (PEG) precipitation to remove the excess staple DNA (Figure 1, left). The removal of excess staples by purification was confirmed using electrophoresis (Figure S1). The resulting DNA origami solution was expected to show a 1:1 ratio of scaffold to staple. AFM observations showed that the yields of the conventional DNA origami and truss origami structures were 90% and 88%, respectively (shown in Figure 2). Without PEG purification, when the sample annealed under the conditions of a 1:1 ratio of scaffold to staple, only a non-structured state was observed (Figure S19). The purified DNA origami solution was then mixed with a solution containing the nonionic surfactant Span80, to encapsulate the DNA origami structures in micron-sized compartments in the form of water-in-oil-droplets. To prepare the compartment, the droplet hydrophobic interface was filled with the surfactant. In the study, without the surfactant, the droplets are immediately fused to make a bulk water phase and oil phase. We confirmed the adsorption of DNA (stained by SYBR gold) to the droplet internal surface by fluorescence imaging. No significant increase in fluorescence intensity was observed in the vicinity of the droplet surface compared with that in the droplet internal space (Figure S3).

The droplets showed different size distributions, depending on the preparation conditions. For the DNA origami structures (without excess staples) encapsulated inside of droplets obtained (Figure 1, upper-center), we performed the re-annealing process using a thermal cycler to first unfold (disassemble) and then re-fold the DNA origami structure (Figure 1, right).

Two types of re-annealing profiles were used after 5 min at 70 °C: a “Normal_45min” profile, performed at −1 °C/min, from 70 °C to 25 °C; and a “Quick_1min” profile, performed at −50 °C/min. We collected these DNA samples by centrifugation, and after PEG purification, we evaluated the morphology of the DNA origami structures using AFM observation (Figures S5–S17). The results are shown in Figure 2. First, purified truss-type origami was used, no significant difference in refolding efficiency was observed between bulk solution conditions and droplet-encased conditions when the “Normal_45min” profile was used. When the “Quick_1min” profile was used, almost no structures were observed under bulk solution conditions, but more than 20% of the folded structure was observed under droplet-encased conditions (Figure 2a). This result is likely due to the diffusion of the minimum necessary DNA staple set at elevated temperatures. In the bulk solution, the reaction volume is on the order of 10^20^ nm^3^, and the indispensable staple set that is necessary for the DNA origami to fold is unlikely to be attracted to the unfolded structure again; however, when the DNA origami is encased within a droplet, the staple set would remain near the scaffold owing to the finite volume of the droplet (~10^10^ nm^3^).

When a purified, conventional DNA origami was used, the refolding efficiency in the bulk solution was found to be extremely low (Figure 2b). This result indicates that the scaffold to staple ratio of 1:1 is disadvantageous for the refolding of the conventional DNA origami, which requires the minimization of inter-strand distances. In the droplet, a higher efficiency was expected, but the results showed 17% refolding under normal annealing conditions, and 5% refolding under quick annealing conditions. It is known that the DNA origami’s structural stability is governed by variations in the melting temperature of the individual staple strands [20]. The difference in the distribution of the melting temperature of the staple DNAs between the two structures is not significantly large (truss origami: 62.2 ± 2.8 °C, conventional origami: 61.8 ± 4.8 °C (Figure S18)). These results demonstrate that the truss origami’s sparse alignment of DNA double helixes is advantageous for refolding compared with the conventional origami with its dense alignment. The yield of conventional DNA origami depends on the complexity of the design model, the arrangement of crossover branches, and the folding path of the scaffold [21,22,23]. The truss origami structure has a truss-like wireframe, with a crossover branch [18,19]. Therefore, compared with conventional DNA origami, the double helix DNA per unit area is designed to be sparse, and the inhibitory effects of the base pairing—due to electrostatic repulsion between DNA strands—are mitigated. The design shows that the angle at which the staple inserts results in a lower energy barrier compared with conventional DNA origami, where the scaffold must turn 180° at the position of the crossover branch.

**Figure 3:**
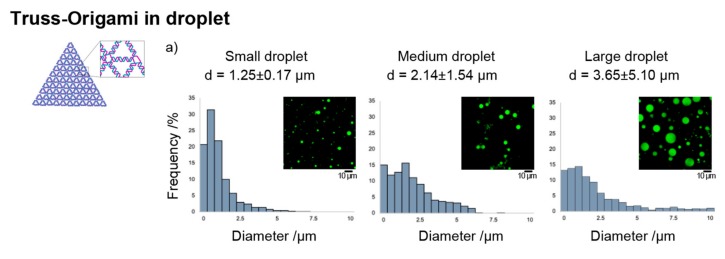

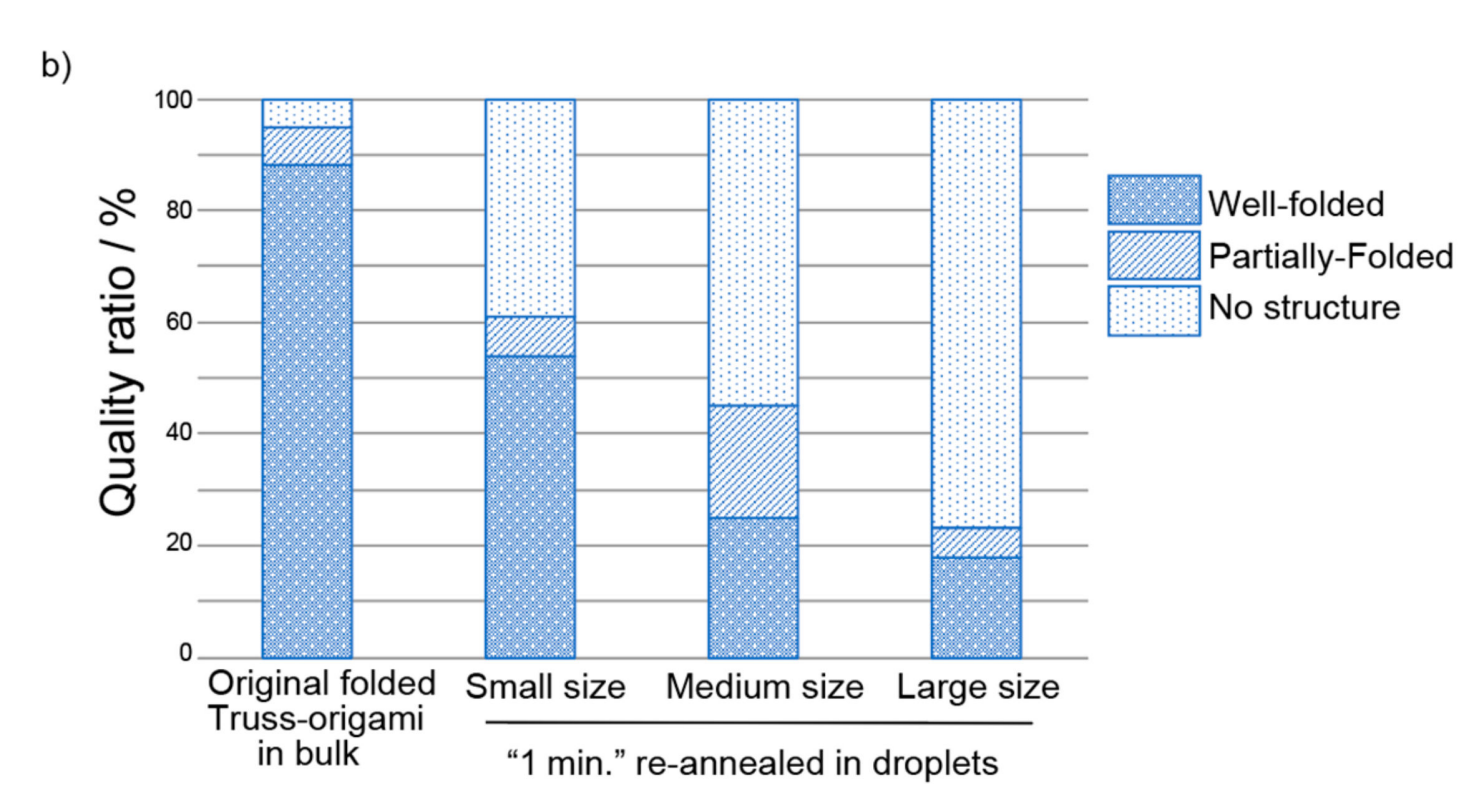
Refolding efficiencies of the DNA truss structures in different sizes of droplets under the “Quick_1min” annealing profile. (**a**) Size distribution of the droplets prepared under the conditions described in the main text (Section 4.3). The value “d” is the average +/− their standard deviations of the diameter of the droplets. (**b**) Refolding efficiencies of the truss origami in the droplets. The criteria for structure formation are the same as in Figure 2. A more efficient (>50%) refolding phenomenon was confirmed with a smaller droplet. The method used to analyze the droplet size distribution is shown in the supporting information (Figure S2).

Next, the effects of quick annealing on the refolding of the truss-DNA origami structure were examined within differing droplet sizes, and the results are shown in Figure 3. In droplets with a diameter of approximately 1 µm, the refolding efficiency exceeded 50%, even under quick annealing conditions with a cooling time of 1 min. In contrast, in droplets with an average diameter exceeding 3 µm, the refolding efficiency was 20% or lower.

## 3. Discussion

**Figure 4:**
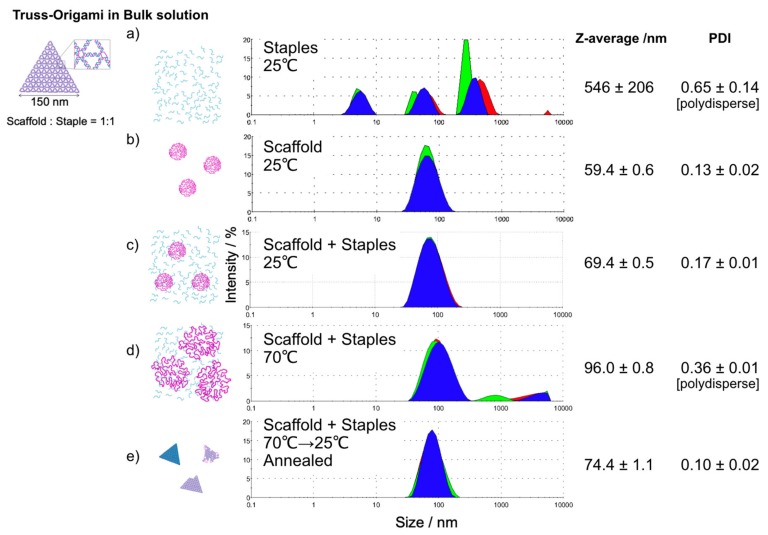
Size distributions before and after the folding of truss-DNA origamis in bulk solution were determined by dynamic light scattering (DLS): the size distributions of (**a**) staple DNA only, (**b**) scaffold DNA only, (**c**) scaffold DNA and staple DNA mixture (scaffold: staple = 1:1), (**d**) the mixture sample with an elevated temperature up to 70 °C and (**e**) the annealed DNA mixture sample. Note that the annealed structure is a mixture of well-folded, partially folded and no structure, as shown in Figure 2. The samples were assessed three times for each (shown as the red, green and blue colors). Staple DNAs showed a polydisperse distribution of their size (polydispersity index (PDI) >0.3), however, just mixing the Scaffold and Staple DNAs showed rather packed state (**c**). The temperature elevating caused a large spread exceeding 1 µm (**d**). The size distributions (1, 2, and 3 µm) of the microcompartment droplets used in this study suggest that physical confinement restricts the spread of DNA molecules observed in (**d**).

Here, we discuss the reasons underlying why the refolding efficiency of origamis increases as the volume decreases. It was difficult to visualize the inside of the droplet, especially the “folding process”, and the intermediate state of folding at high temperatures using AFM. DNA origami folding is a phenomenon in which the spread of the scaffold and the staple DNA mixture becomes compact, so we confirmed the size distributions of the DNA mixture solution as particles in the bulk solution through dynamic light scattering (DLS) (Figure 4). By cumulant method, their z-average and poly-dispersity index (PDI) was analyzed from the intensity data. In particular, the difference in the polydisperse index before and after folding should be noted. In general, PDI indicates the deviation from the monodispersity. For example, the scaffold and annealed structures are represented as monodisperse (Figure 4b,e), while the staple DNA set is represented as polydisperse (Figure 4a). Interestingly, the mixture of the scaffold and staple set at 25 °C shows monodispersity (Figure 4c). It should be noted that the structure is not one kind just because it is monodisperse. The annealed structure (shown in Figure 4e) is a mixture of well-folded, partially folded and no structure, as shown in Figure 2. At 70 °C, the broad distribution pattern and polydispersion condition were observed (Figure 4d). Considering droplets with diameters of approximately several micrometers, the DNA mixture appears to be forcibly trapped inside the compartment, unlike the natural distribution of the flexible DNA chains mixture (with distribution areas larger than micrometers in diameter, Figure 4d in the bulk solution at 70 °C). Essential staples are kept inside of the droplet and, in addition, the scaffolds and staples might be forced to occupy denser conditions than their natural distribution, owing to the spatial constraints of the droplet and the inhibition of structure formation based on electrostatic repulsion being suppressed, thereby promoting refolding. Thus, because of the aforementioned physical reasons, the efficiency of DNA origami refolding in microcompartments must be higher than that in bulk solution. We also attempted to construct smaller, sub-micron-sized droplets [24] for refolding experiments. However, sub-micron droplets enclosing DNA origamis and precursor mixture could not be constructed. The reason for this might be because the DNA mixture occupies a rather large space compared to the null droplet space. The development of a method for providing nano compartments in a quantitative manner is necessary.

Does the chemical condition of the droplet surface affect the refolding process? Fallah-Araghi et al. reported that, in a simple organic synthesis reaction using a droplet to reversibly generate a fluorescent imine from an aldehyde and an amine, the amount of imine generated increased as the reaction field volume decreased [14]. They showed that the amount of imine generated increased in smaller droplets with larger specific interfacial areas because the adsorption and desorption of molecules at the droplet interface promoted the synthesis of imines. Yanagisawa et al. recently used a gelatin-encapsulated lipid droplet to promote a folding transition, from a random coil structure to a beta-sheet structure, in a size-dependent manner, particularly for droplets with diameters of 50 µm or less, rather than a bulk gel. They clarified that a microgel structure with a large Young’s modulus can occur [17], and they cited an increase in the lipid specific surface area as a contributing factor. In addition, Kato et al. and Pasquale et al. reported an increase in the protein synthesis rate as the compartment size decreased in a droplet containing a cell-free transcription and translation system. They also described the effects of the membrane surface, due to differences in lipid composition [13,16]. In a study in which a chemical oscillator using a DNA logic circuit was operated in a droplet, an oscillating reaction was observed under droplet conditions [25]. The authors, Weitz et al., considered that the observed oscillating behavior may be due to the bias of the number of constituent molecules contained by the DNA logic circuit and the specific adsorption of the enzyme onto the lipid membrane. Recently, Sato et al. reported driving a DNA-amplification circuit inside of the lipid unilamellar vesicle. They suggested that the differences between amplification in test tubes and GUVs are largely due to an interaction between lipid membranes and DNAs [26].

In this study, droplet conditions may promote DNA origami refolding due to the nonionic oil-water interface, which does not attach to DNA molecules and may increase the effective concentration of the DNA mixture. Fluorescence microscopy observation produces a 100 nm level blurring effect, even for a single molecule; therefore, further analysis was difficult due to the limitations of imaging resolution. When charged phospholipids or surfactants are used, the encapsulation efficiency for DNA mixtures and/or the surface effect on the refolding kinetics might be controlled. Such changes in lipid composition are beyond the scope of this paper, but should be considered for future research focusing on the effects of finite volumes on DNA origami refolding.

## 4. Materials and Methods

### 4.1. Materials

All staple strands of ssDNA were purchased from Eurofins Genomics Tokyo (Tokyo, Japan). Single-stranded M13mp18 viral DNA (as scaffold ssDNA) was purchased from Tilibit Nanosystems (Garching, Germany). The nonionic surfactant Span80 (Sorbitan, mono-(9*Z*)-9-octadecenoate) and liquid paraffin were purchased from Wako Pure Chemical Industries, Ltd. (Tokyo, Japan). All chemicals were used without further purification.

### 4.2. DNA Origami Design and Preparation

The conventional DNA origami structure was designed using the caDNAno software [27]. The truss-DNA origami structure was designed using the vHelix software [28]. The designs are described in the supporting information (Figure S4). The assembly of the origami structures was accomplished by mixing 1 nM M13mp18 scaffold DNA with 10 nM staple strands in 25 µL of TAE-Mg buffer containing 40 mM Tris-acetate (pH 8.3), 1 mM EDTA and 12.5 mM MgCl_2_. The mixture was annealed as follows: (1) heating at 70 °C for 5 min; (2) cooling from 70 °C to 25 °C at a rate of −0.1 °C/min; (3) cooling and storage at 25 °C. The assembled structure was purified by PEG-precipitation [29]. The resultant concentration of the DNA sample was estimated from electrophoresis band intensity (Figure S1) using ImageJ software.

### 4.3. Preparation of Droplets Containing DNA Origami

First, as an oil phase, 5% (*v*/*v*) Span80 was sufficiently dissolved in liquid paraffin. Then, the DNA origami solution was added and stirred on a vortex machine (PresentMixer2013, 2,800 rpm, Titec Corp., Saitama, Japan) to prepare a water-in-oil emulsion (droplets). The mixture volume was comprised of 100 µL DNA solution with 900 µL paraffin to obtain a medium-sized droplet. Although the distributions are non-uniform (Figure S2), the typical droplet size was arranged by changing the ratio between the DNA solution and oil phase as well as the vortex time as follows: small size, 2.5% (*v*/*v*), vortexed 5 min; medium size, 10% (*v*/*v*), vortexed 10 min; and large size, 10% (*v*/*v*), vortexed 15 sec.

### 4.4. DNA Sample Extraction from Droplets

To observe DNA nanostructures by AFM or electrophoresis, the DNA solution must be extracted from the droplets. The procedure is described below. First, the hydrophilic contents of the droplets in the microtube were collected at the bottom of the tube by centrifuging the emulsion at 20,000 × g for 10 min. Second, after removing the upper oil phase, the residual droplets were dispersed onto the microtube wall by tapping or vortexing the tube vigorously. The dispersed droplet contents were collected by centrifugation, again. By repeating this process, a sufficient quantity of DNA solution (approximately 20 µL) was obtained for observation.

### 4.5. Agarose Gel Electrophoresis

The samples were loaded onto a 1.0% agarose gel, containing 5 mM MgCl_2_, in a 0.5 × TBE (Tris-borate-EDTA) buffer solution (pH 8.0), for electrophoresis at 50 V, at 4 °C. The gels were then imaged by ChemiDOC MP (Bio-Rad Laboratories, Inc., Hercules, CA, USA), using SYBR^®^ Gold Nucleic Acid Gel Stain (Thermo Fisher Scientific, MA, USA) as the staining dye.

### 4.6. AFM Observation

AFM imaging was performed using Nano Live Vision (RIBM, Tsukuba, Japan). Typically, a drop (2 µL) of the sample (5 nM) was deposited onto a freshly cleaved mica surface. After 1 min incubation, the surface was rinsed with 10 µL TAE-Mg buffer, and then scanned in approximately 120 µL TAE-Mg buffer using a small cantilever (9 µm long, 2 µm wide and 130 nm thick; BL-AC10DS, Olympus, Tokyo, Japan) with a spring constant of approximately 0.1 N/m and a resonant frequency of approximately 300–600 kHz in water. Typically, 320 × 240-pixel images were obtained, at a scan rate of 0.2 frames per second. AFM images were analyzed using the AFM Scanning System Software (Olympus).

### 4.7. DLS Measurement

DLS measurements were performed with a Zetasizer nano ZSP (Malvern, Tokyo, Japan). The data was evaluated by the cumulant method for obtaining z-average and PDI values. For the DLS analysis, the DNA samples needed to be concentrated for overcoming the detective limit. DNA solution samples were adjusted to a concentration of 50 nM staples (Figure 4a), 50 nM scaffold (Figure 4b) or 30 nM scaffold with 30 nM staples (Figure 4c–e) in TAE-Mg buffer. The buffer solution was filtered through hydrophilic filters, with a pore size of 0.22 µm (Merck Millipore, Billerica, MA, USA), before use. A DNA sample volume of 12 µL was transferred into quartz light scattering cuvettes for measurement. The measurements were performed at 25 °C or 70 °C.

## 5. Conclusions

In this study, the DNA origami refolding process in the microcompartment occurred, even at a scaffold: staple ratio of 1:1. The truss origami refolding appeared to be more effective than the conventional DNA origami structure. Even the quick annealing conditions of one minute provided an approximately 50% refolding yield by using a microdroplet compartment of approximately 1 µm in diameter. DLS measurements showed that the natural distribution of the unfolded DNA mixture was larger than the diameters of the microcompartment droplets. Studying how dynamic state biopolymers change in response to the physicochemical microenvironment to which the biopolymers are exposed is essential for understanding the principles of life phenomena, and for the production of novel nanostructures. Currently, DNA nanotechnology is attracting a great deal of interest due to its exceptionally high design flexibility and feasibilities [5,6,7]. This study could lead to the determination of specific conditions that are advantageous for the production and maintenance of artificially designed molecules, such as molecular machines or micron-sized molecular robots [30,31,32].

## Supplementary Materials

The following are available online at https://www.mdpi.com/1420-3049/25/1/8/s1, Figure S1: Electrophoresis data for the DNA origami. Figure S2: Analyzing the size distribution of the droplets. Figure S3: Fluorescence microscopic images of the droplets, including DNA mixtures stained with SYBRGold. Figure S4: Designs of the DNA origamis used in this study. Figure S5. AFM images of the truss origami from the opened droplets. Figure S6. AFM image set of the purified conventional DNA origami structures (before re-annealing). Figure S7. AFM image set of the conventional DNA origami structures in bulk solution with a “Normal_45min” annealing profile. Figure S8. AFM image set of the conventional DNA origami structures in “medium”-sized droplet with a normal “Normal_45min” annealing profile. Figure S9. AFM image set of the conventional DNA origami structures in bulk solution under “Quick_1min” annealing conditions. Figure S10. AFM image set of the conventional DNA origami structures in “medium”-sized droplets under “Quick_1min” annealing conditions. Figure S11. AFM image set of the purified DNA truss structures (before re-annealing). Figure S12. AFM image set of the DNA truss structures in bulk solution with a “Normal_45min” annealing profile. Figure S13. AFM image set of the DNA truss structures in “medium”-sized droplet with a “Normal_45min” annealing profile. Figure S14. AFM image set of the DNA truss structures in bulk solution under “Quick_1min” annealing conditions. Figure S15. AFM image set of the DNA truss structures in “small”-sized droplets under “Quick_1min” annealing conditions. Figure S16. AFM image set of the DNA truss structures in “medium”-sized droplets under “Quick_1min” annealing conditions. Figure S17. AFM image set of the DNA truss structures in “large”-sized droplets under “Quick_1min” annealing conditions. Figure S18. Distributions of the melting temperature of the staple DNA set. Table S1: DNA sequences of the staples used for the truss DNA origami. Table S2: DNA sequences of the staples used for the conventional DNA origami. Figure S19. The “folding” efficiencies of DNA origami from a mixture of a 1:1 ratio of the scaffold to staple (1 nM).
