# Supplementary material for: DNA Origami “Quick” Refolding inside of a Micron-Sized Compartment
Source: Molecules. 2019 Dec 18;25(1):8. doi: 10.3390/molecules25010008 (PMC6983259; doi:10.3390/molecules25010008)
Supplement: Supplementary file 1 [file molecules-25-00008-s001.pdf]

molecules

MDPI

Supplementary materials

DNA origami "Quick" refolding inside of a micron-sized compartment

Taiki Watanabe[1], Yusuke Sato[1,2], Hayato Otaka[1], Ibuki Kawamata[1], Satoshi Murata[1] and Shin-ichiro M. NOMURA[1,*]

[1] Department of Robotics, Graduate School of Engineering, Tohoku University, Sendai 980-8579, Japan
[2] Department of Computer Science, Tokyo Institute of Technology, Kanagawa 226-8502, Japan
* Correspondence: nomura@molbot.mech.tohoku.ac.jp; Tel.:+81-22-795-6910 (S.-i.M.N.)

Contents:

Figure S1: Electrophoresis data for the DNA origami.

Figure S2: Analysing the size distribution of the droplets.

Figure S3: Fluorescence microscopic images of the droplets, including DNA mixtures stained with SYBRGold.

Figure S4: Designs of the DNA origamis used in this study.

Figure S5. AFM images of the Truss-origami from the opened droplets.

Figure S6. AFM image set of the purified conventional DNA origami structures (before re-annealing).

Figure S7. AFM image set of the conventional DNA origami structures in bulk solution with a "Normal_45min"annealing profile.

Figure S8. AFM image set of the conventional DNA origami structures in “medium”-sized droplet with a normal "Normal_45min" annealing profile.

Figure S9. AFM image set of the conventional DNA origami structures in bulk solution under "Quick_1min" annealing conditions.

Figure S10. AFM image set of the conventional DNA origami structures in “medium”-sized droplets under "Quick_1min" annealing conditions.

Figure S11. AFM image set of the purified DNA truss structures (before re-annealing).

Figure S12. AFM image set of the DNA truss structures in bulk solution with a "Normal_45min" annealing profile.

Figure S13. AFM image set of the DNA truss structures in “medium”-sized droplet with a "Normal_45min" annealing profile.

Figure S14. AFM image set of the DNA truss structures in bulk solution under "Quick_1min" annealing conditions.

Figure S15. AFM image set of the DNA truss structures in “small”-sized droplets under "Quick_1min" annealing conditions.

Figure S16. AFM image set of the DNA truss structures in “medium”-sized droplets under "Quick_1min" annealing conditions.

Figure S17. AFM image set of the DNA truss structures in “large”-sized droplets under "Quick_1min" annealing conditions.

Figure S18. Distributions of the melting temperature of the staple DNA set.

Table S1: DNA sequences of the staples used for the truss DNA origami.

Table S2: DNA sequences of the staples used for the conventional DNA origami.

Figure S19. The “folding” efficiencies of DNA origami from a mixtue of a 1:1 ratio of the scaffold to staple (1 nM).

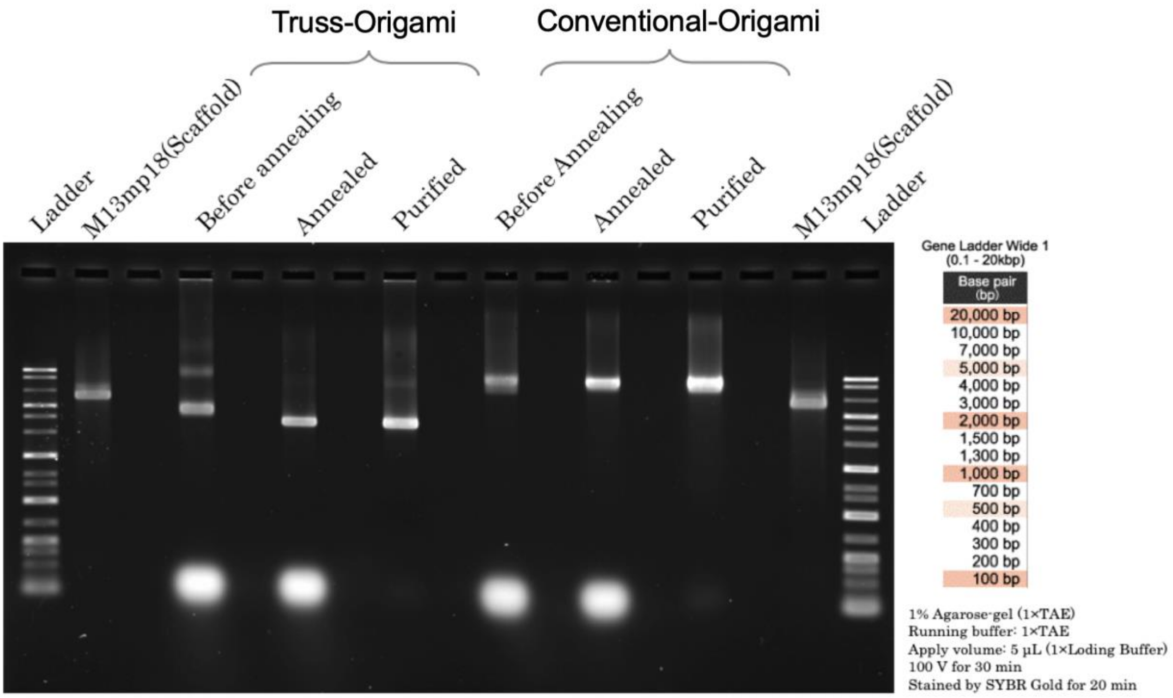

Figure S1. Electrophoresis data for the DNA origamis before and after folding as well as after purification. The concentration of the eliminated non-bounded staples, through purification, was estimated by digital analysis of the band intensity of the electrophoresis image using ImageJ software. We concluded that at least 92% of the non-bounded staples are eliminated (Truss-Origami: 8.0%, Conventional-Origami: 7.2%).

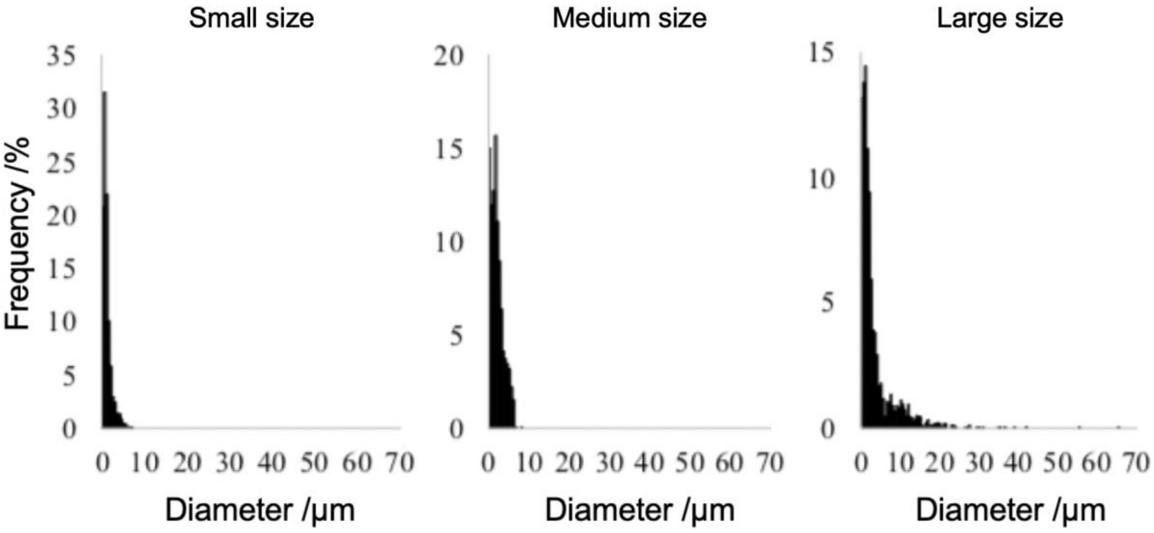

Figure S2. Size distribution of the droplets used in Fig. 3 shown in a wider range. The hydrophilic fluorescent dye, 0.1 mM calcein (Dojindo, Tokyo, Japan), in TAE-Mg buffer was encapsulated inside of droplets using the conditions described in the main text (section 4.3). Cross-sectional fluorescent images of the droplets were obtained using a confocal microscope FV-1000 (Olympus, Tokyo, Japan). The particle size distribution of the droplets was measured in the obtained images using ImageJ software.

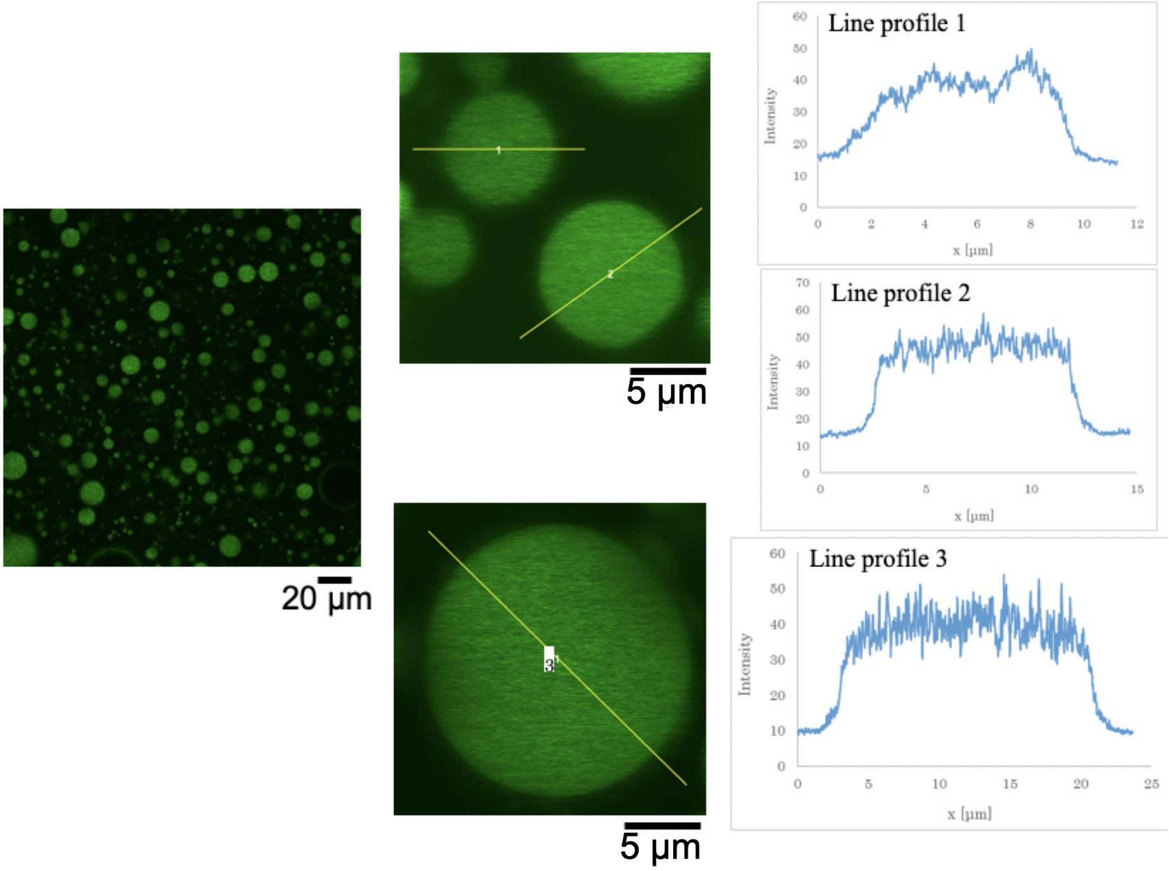

Figure S3. Fluorescence microscopic images and the line-profiles of the droplets, including scaffold DNA (M13mp18, stained by SYBRGold). The images were obtained using confocal microscopy (FV-1000, OLYMPUS, Tokyo, Japan). Laser: Wavelength 473 nm, intensity 7%, PMT Voltage: 500 V. Large droplets were chosen based on to the resolution limits of fluorescent microscopy.

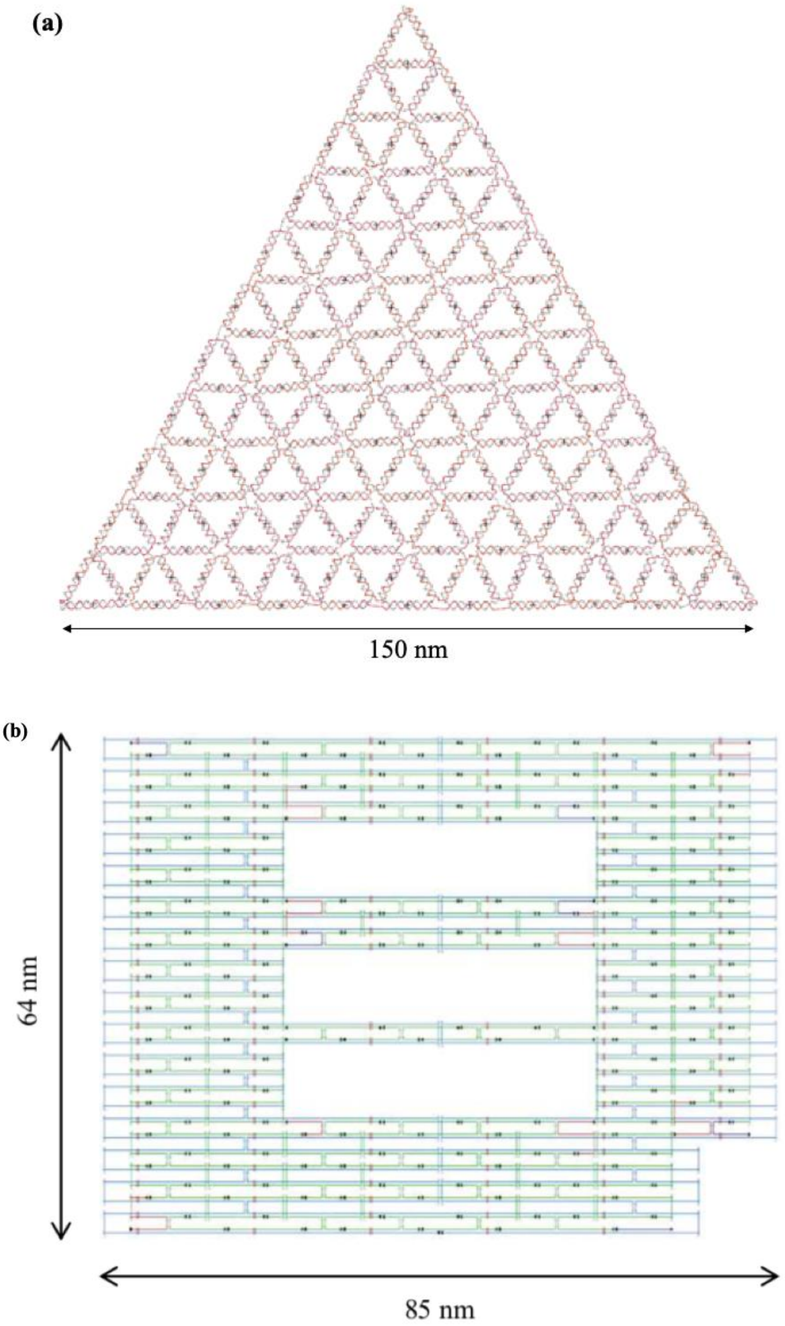

Figure S4. Designs of the DNA origamis used in this study. (a) Truss-DNA origami and (b) conventional DNA origami. The staple sequence data for the Truss and conventional DNA origami structures are shown in Tables S1 and S2, respectively.

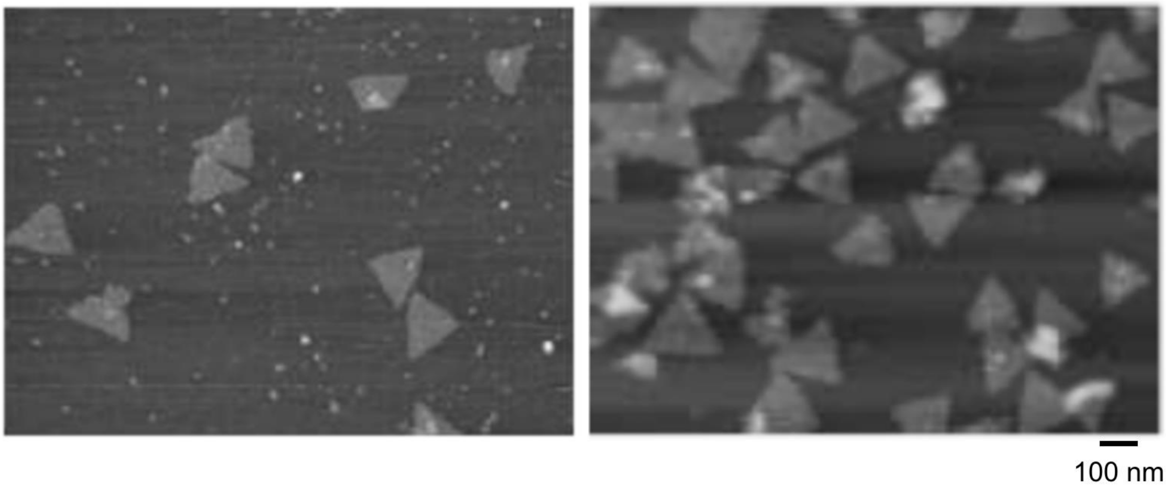

Figure S5. AFM images of the Truss-origami from the opened droplets. Left: Before PEG purification. Right: After PEG purification. The debris appearing as small white dots in the left panel were eliminated after PEG purification.

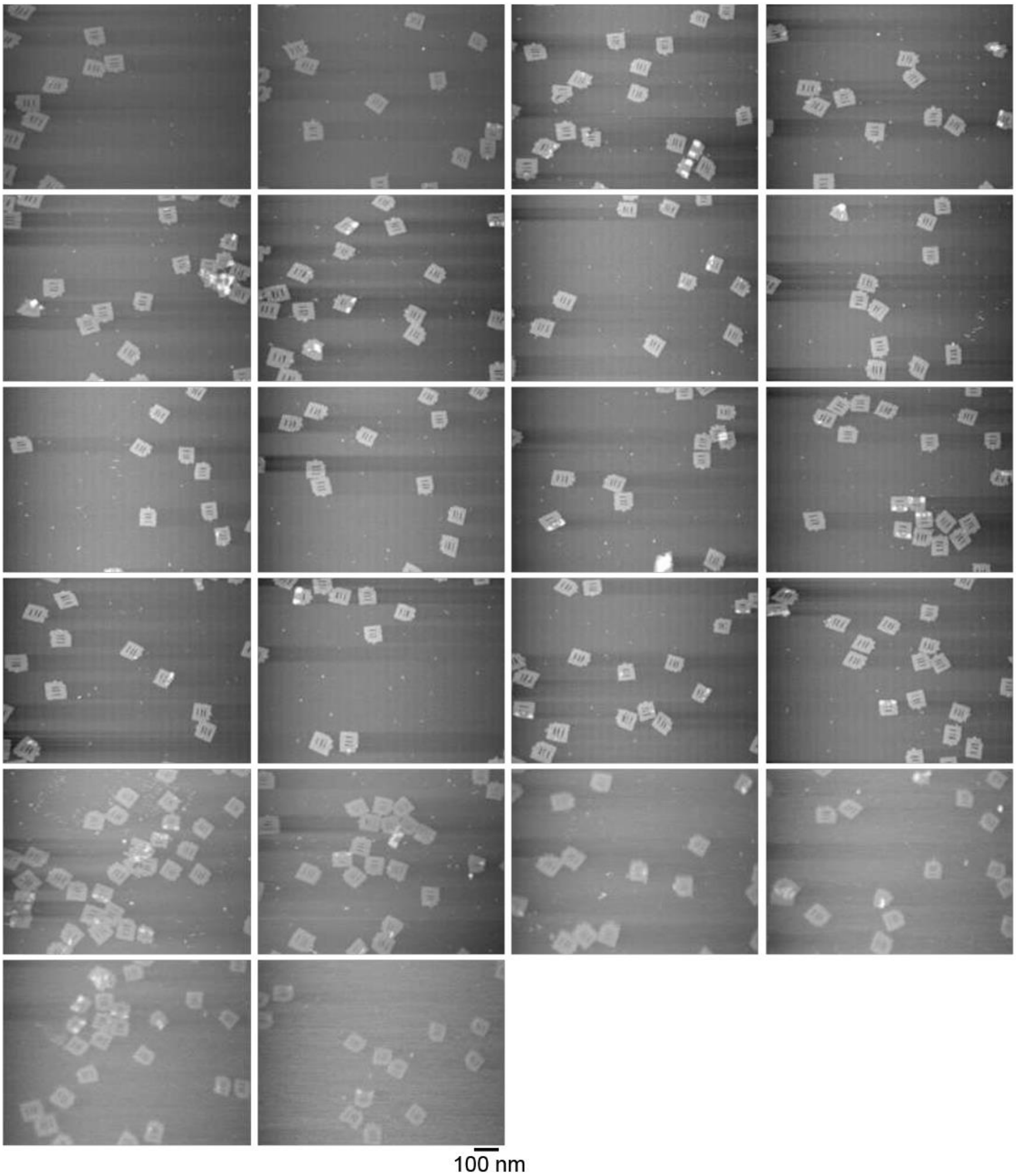

Figure S6. AFM image set of the purified conventional DNA origami structures (before re-annealing).

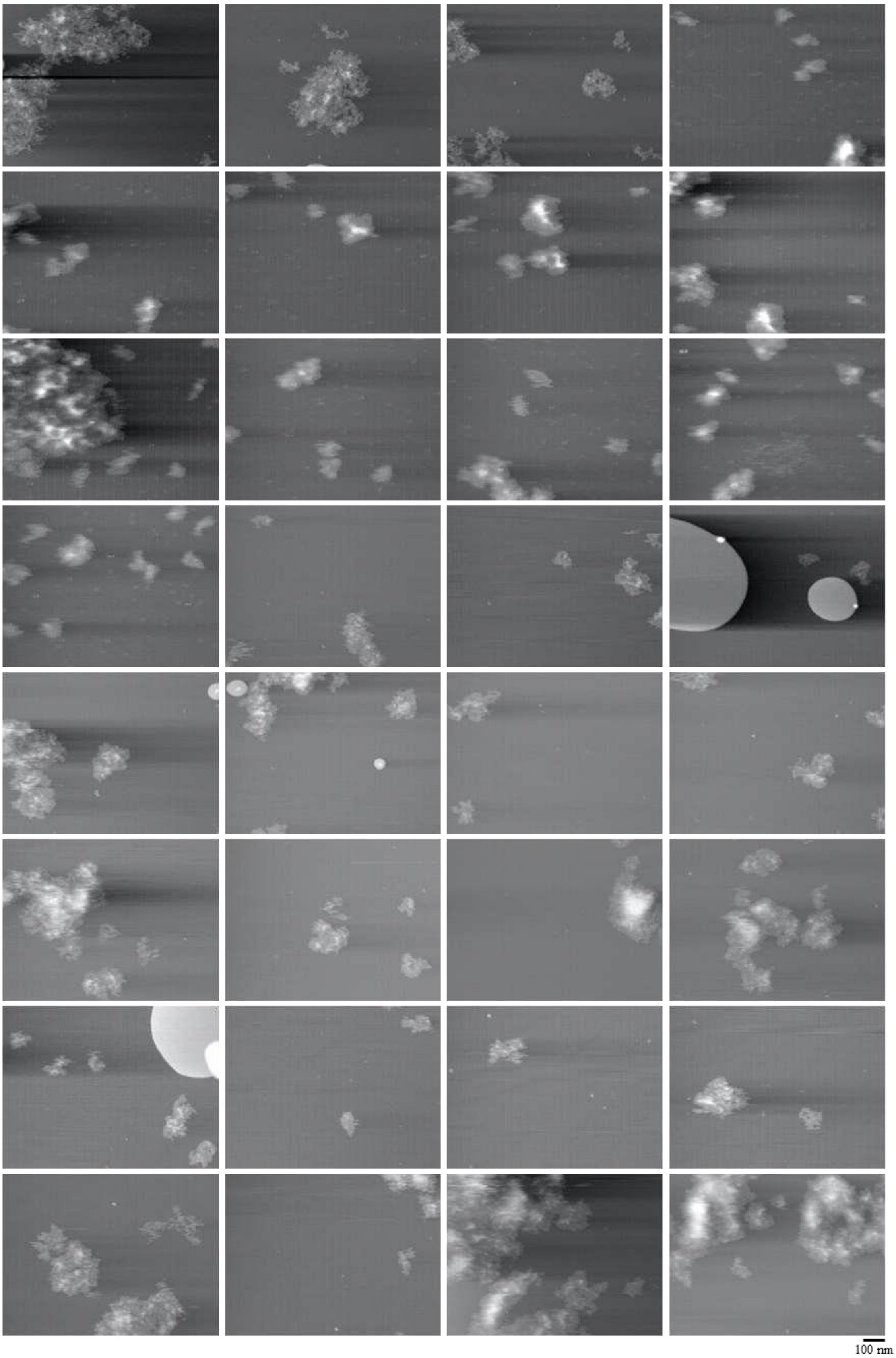
100 nm

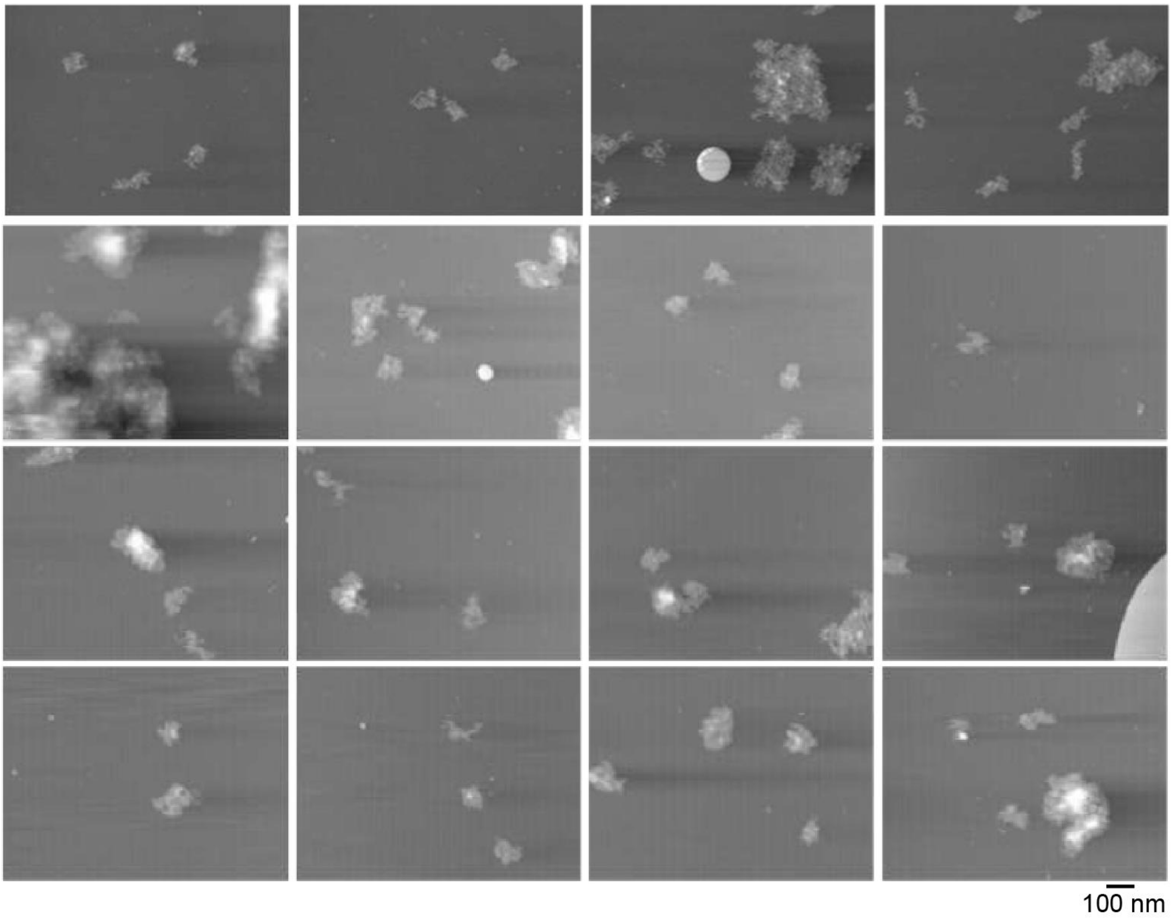

Figure S7. AFM image set of the conventional DNA origami structures in bulk solution with a "Normal_45min"annealing profile.

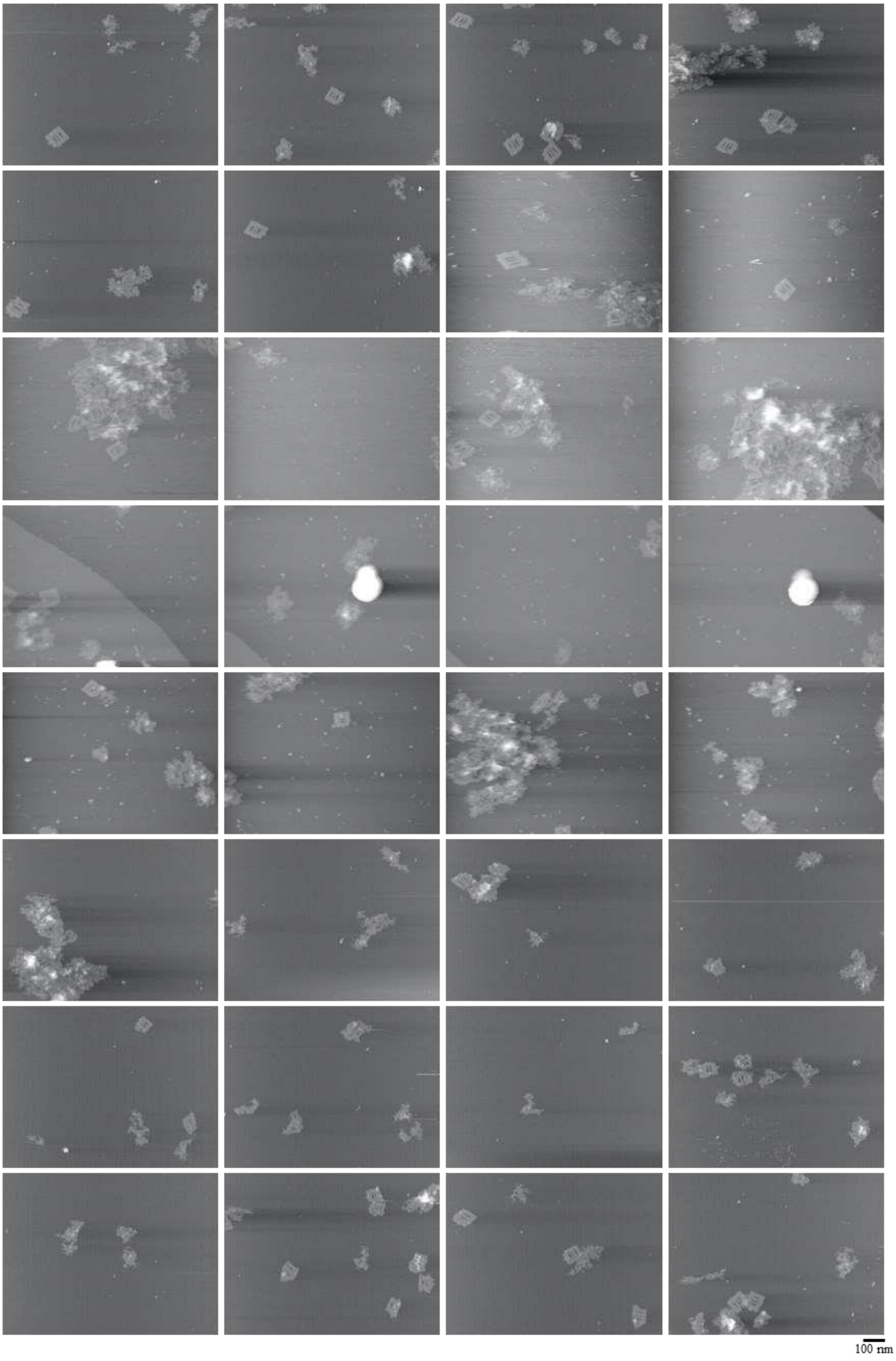
100 nm

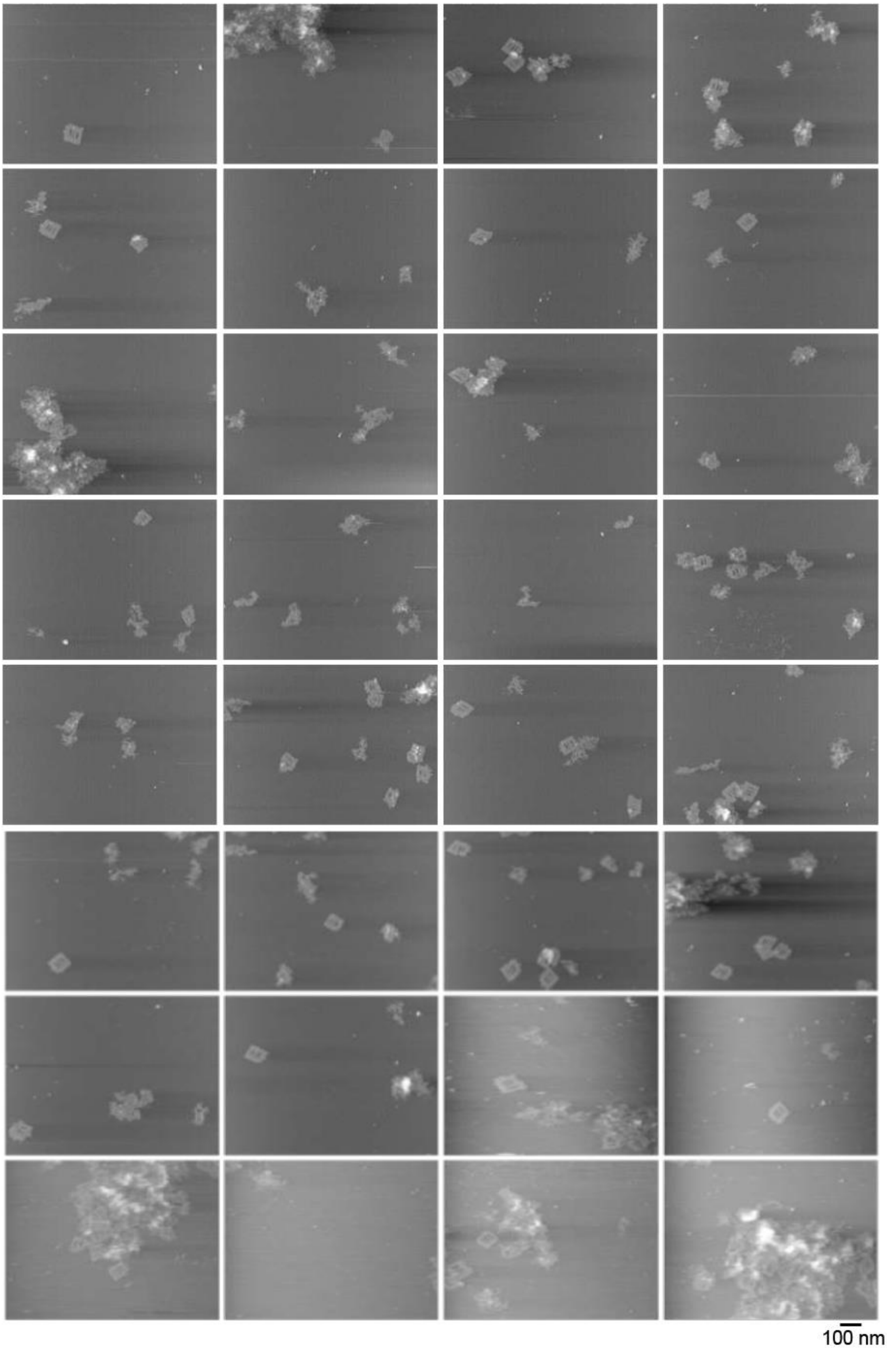

Figure S8. AFM image set of the conventional DNA origami structures in "medium"-sized droplet with a normal "Normal_45min" annealing profile.

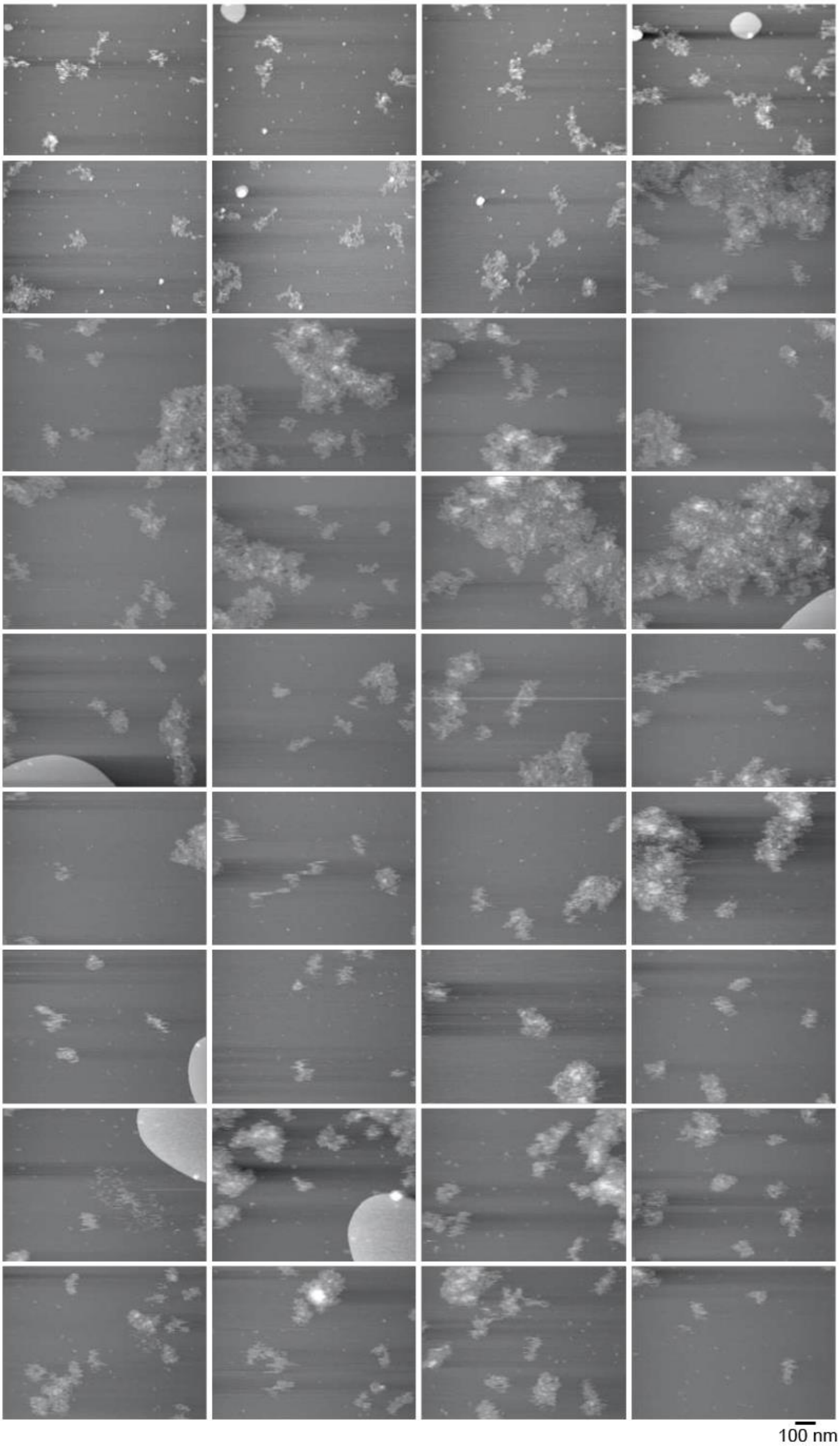

Figure S9. AFM image set of the conventional DNA origami structures in bulk solution under "Quick_1min" annealing conditions.

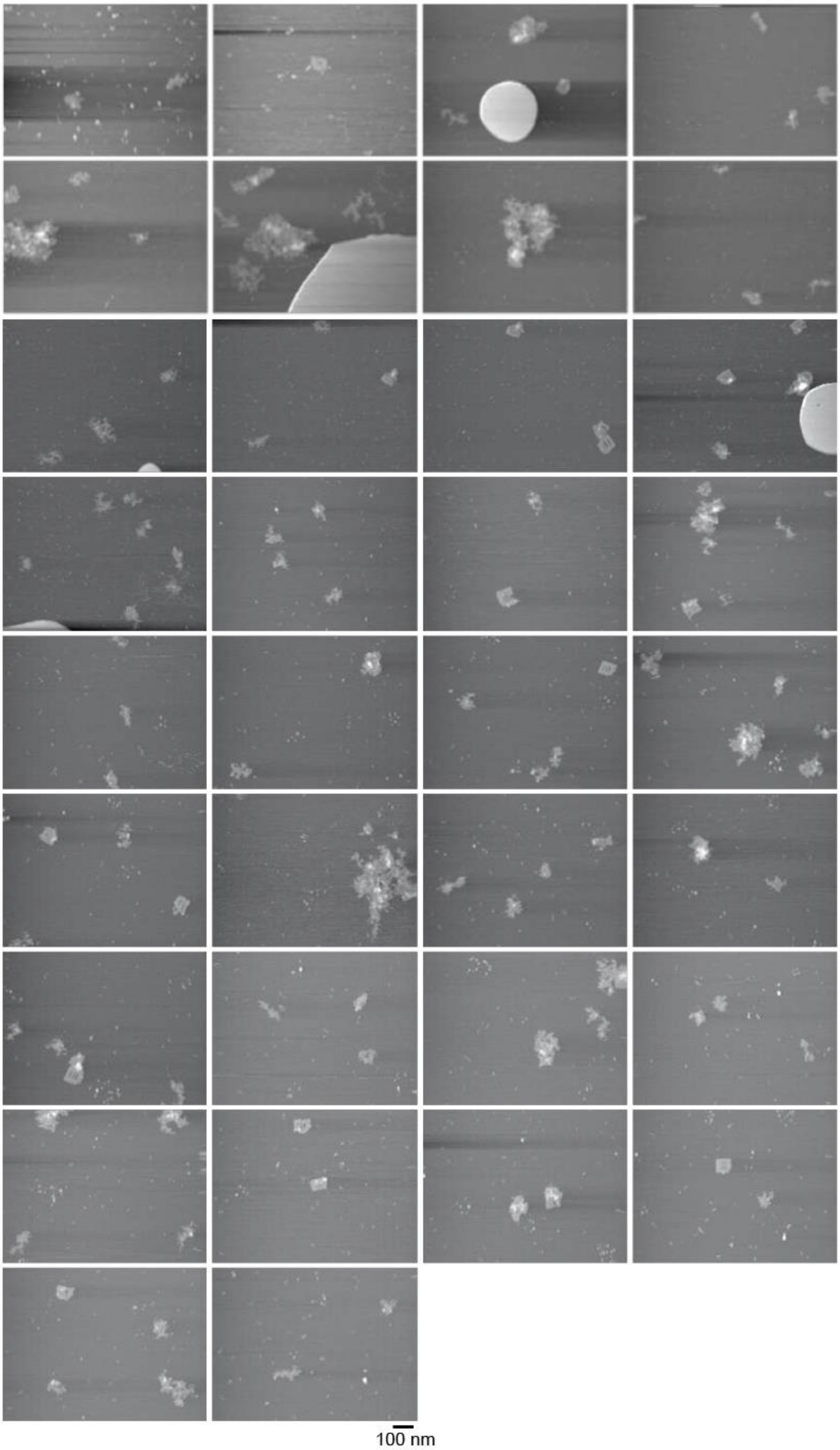

Figure S10. AFM image set of the conventional DNA origami structures in “medium”-sized droplets under "Quick_1min" annealing conditions.

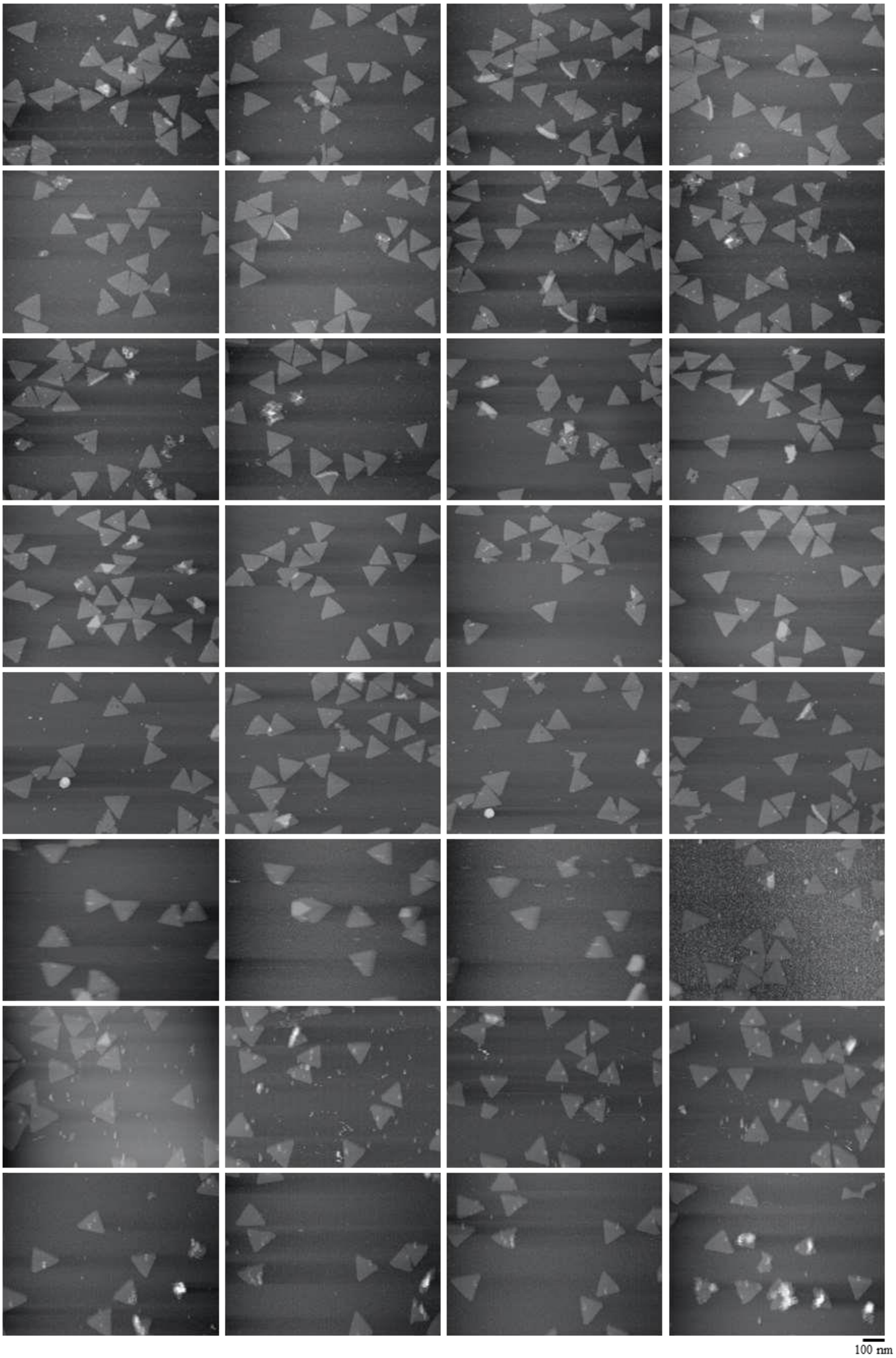
100 nm

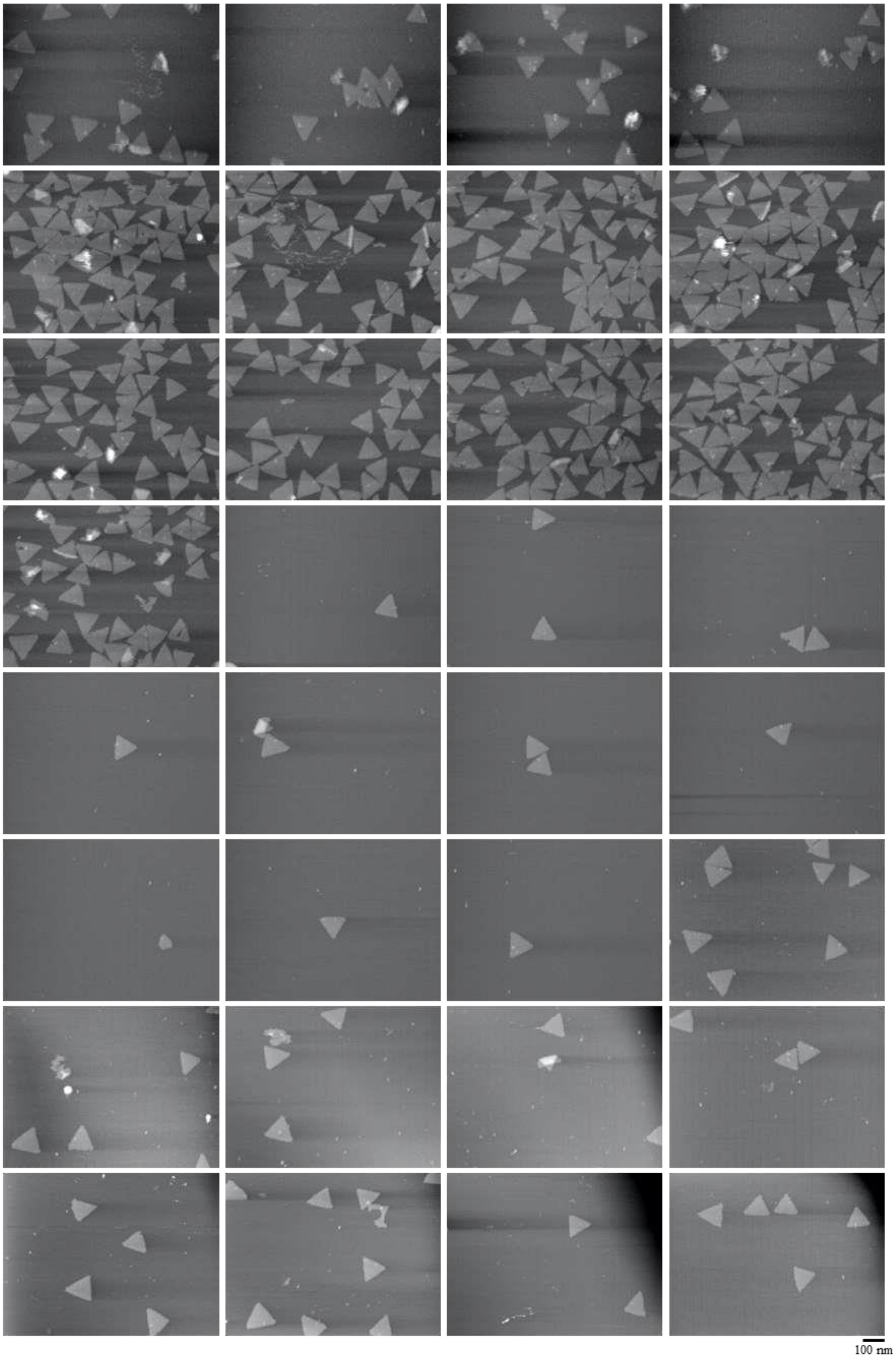
100 nm

138
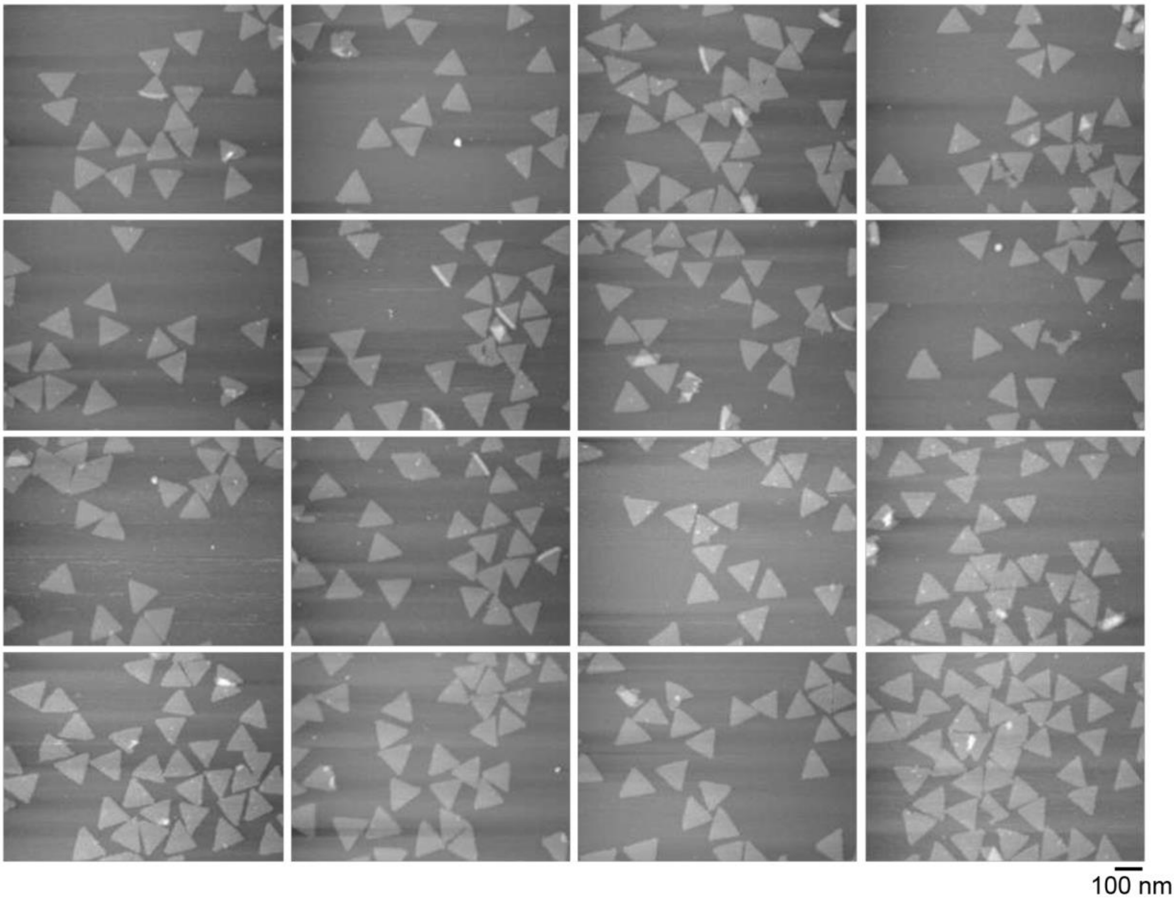

139 **Figure S11.** AFM image set of the purified DNA truss structures (before re-annealing).

140

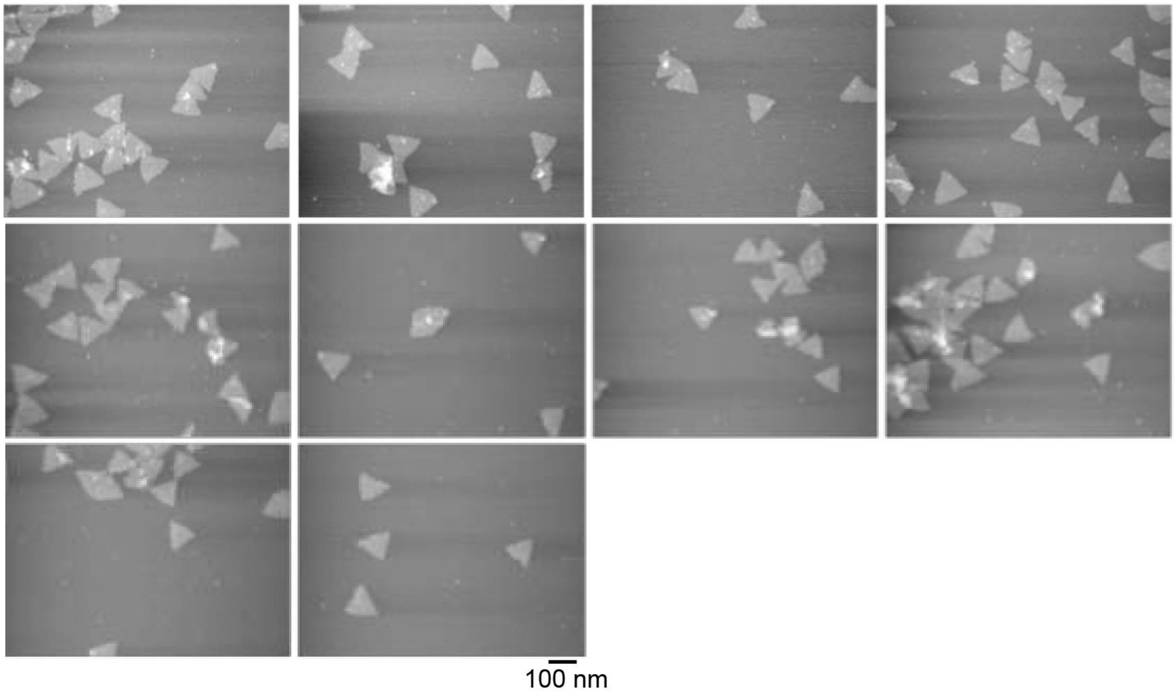

Figure S12. AFM image set of the DNA truss structures in bulk solution with a "Normal_45min" annealing profile.

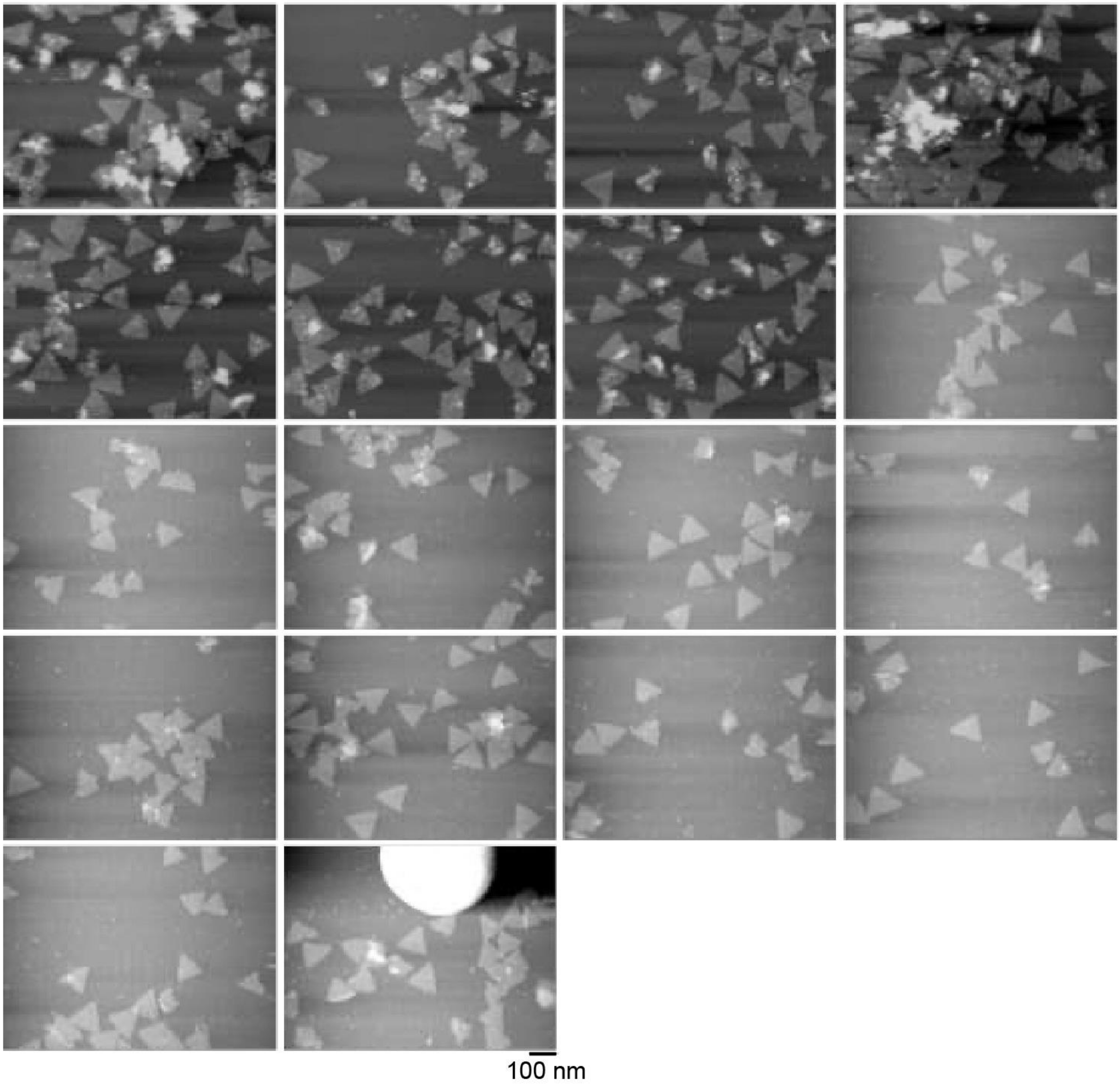

Figure S13. AFM image set of the DNA truss structures in "medium"-sized droplet with a "Normal_45min" annealing profile.

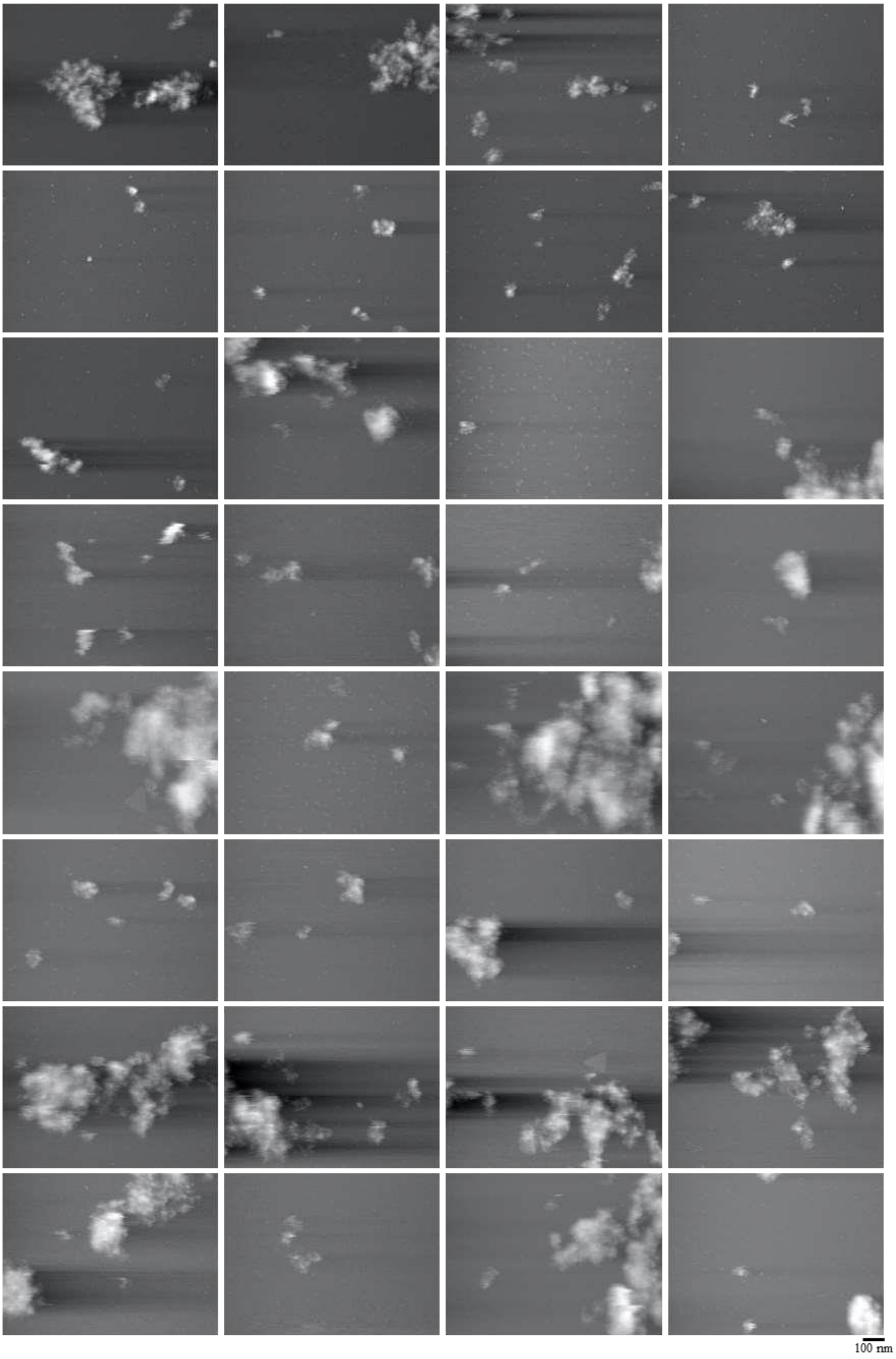

151

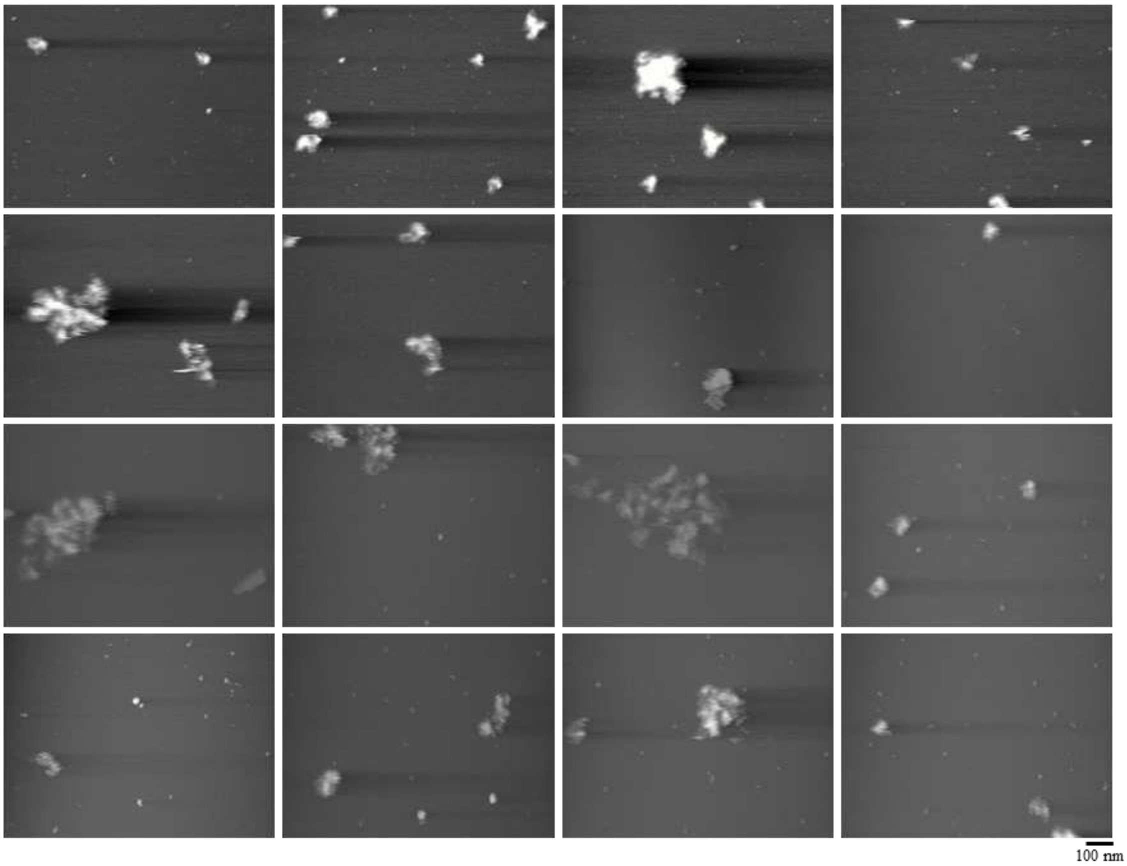

Figure S14. AFM image set of the DNA truss structures in bulk solution under "Quick_1min" annealing conditions.

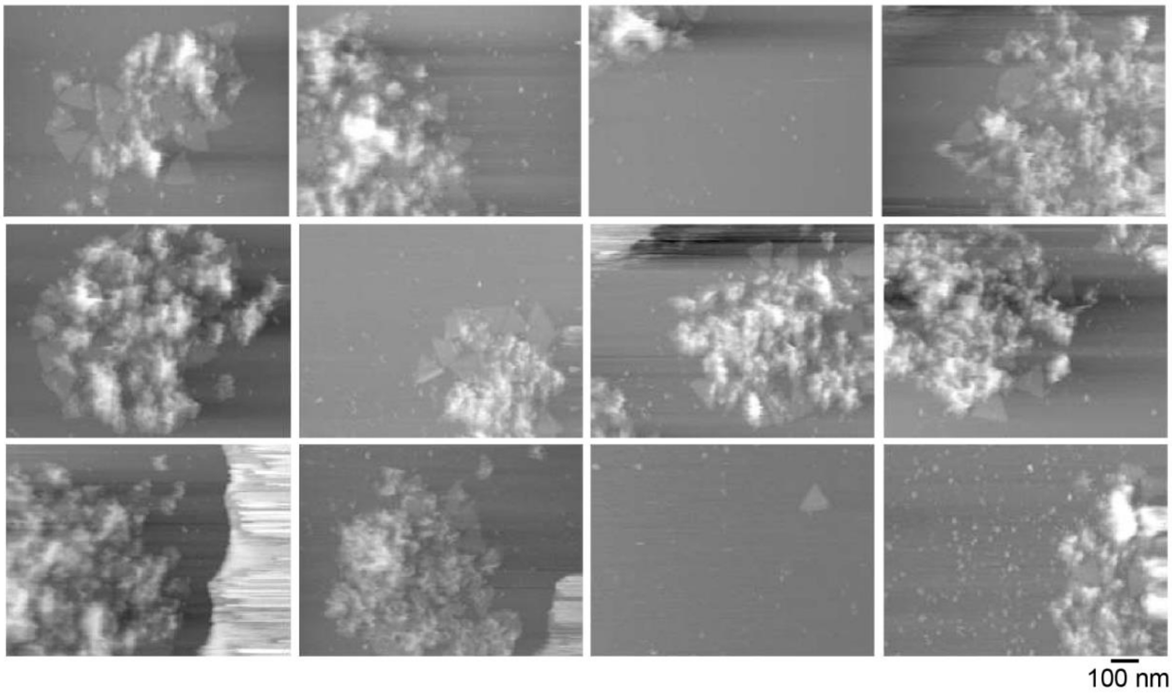

Figure S15. AFM image set of the DNA truss structures in “small”-sized droplets under "Quick_1min" annealing conditions.

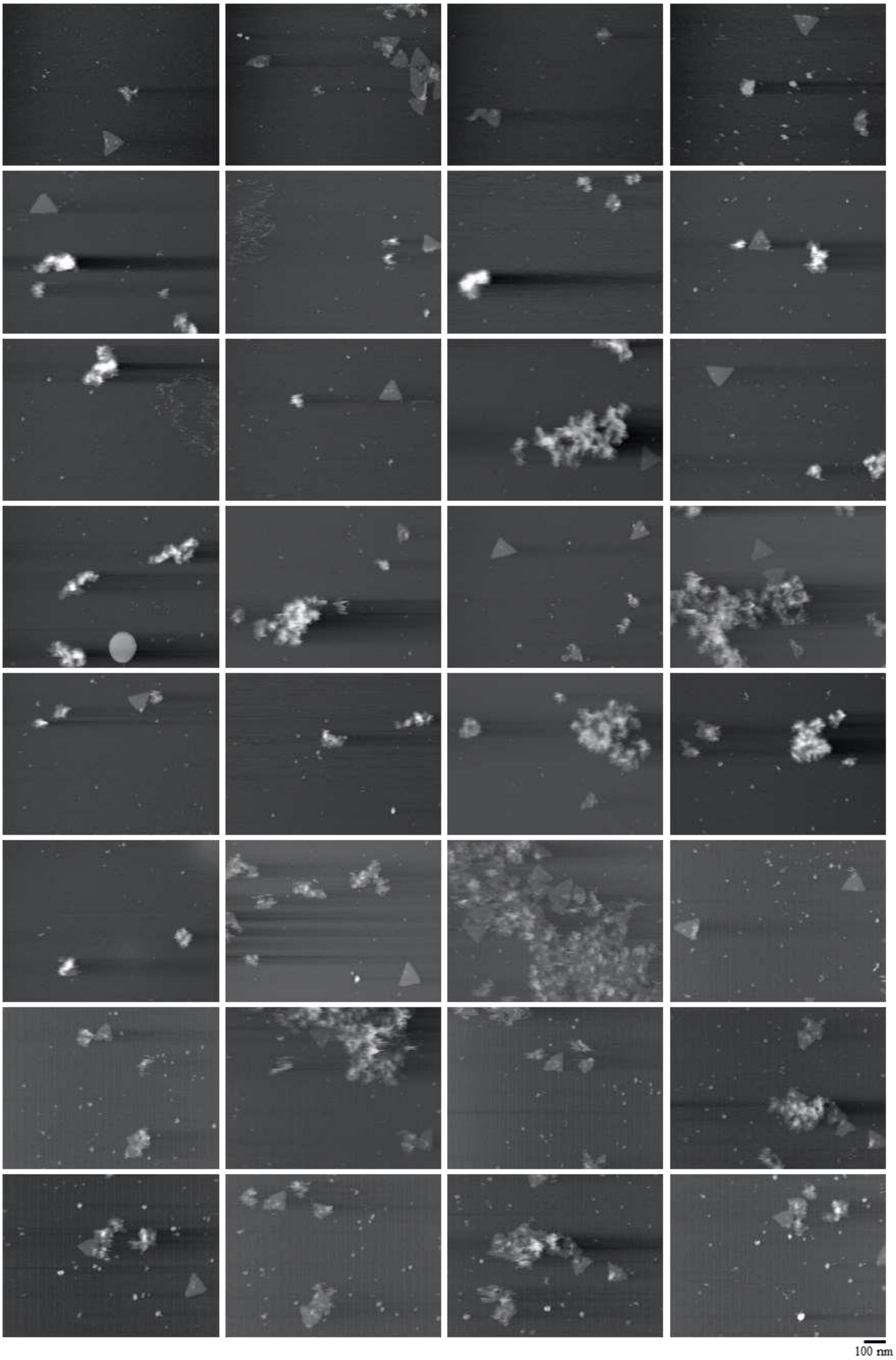
100 nm

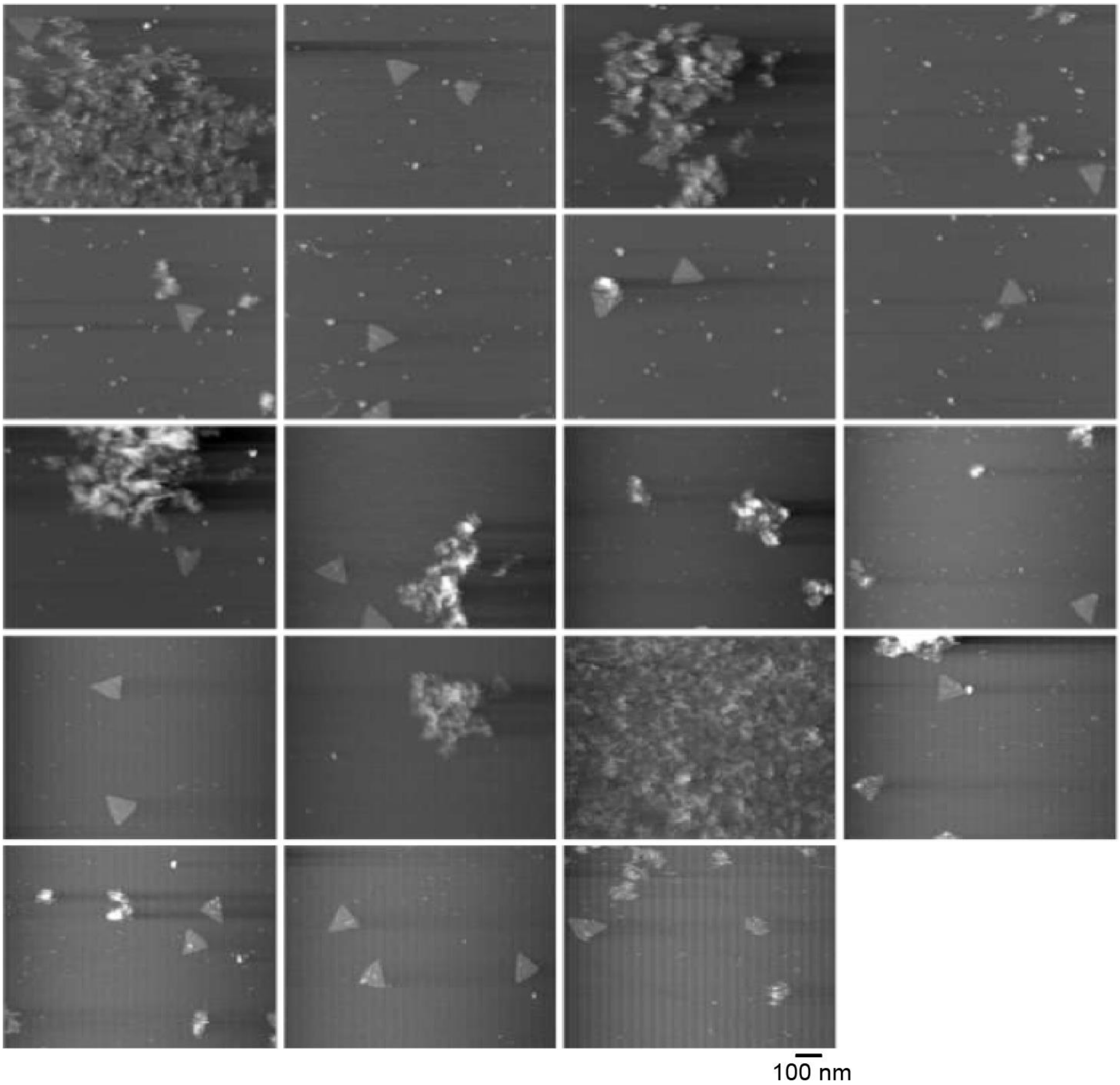

164

165 **Figure S16.** AFM image set of the DNA truss structures in "medium"-sized droplets under
166 "Quick_1min" annealing conditions.

167

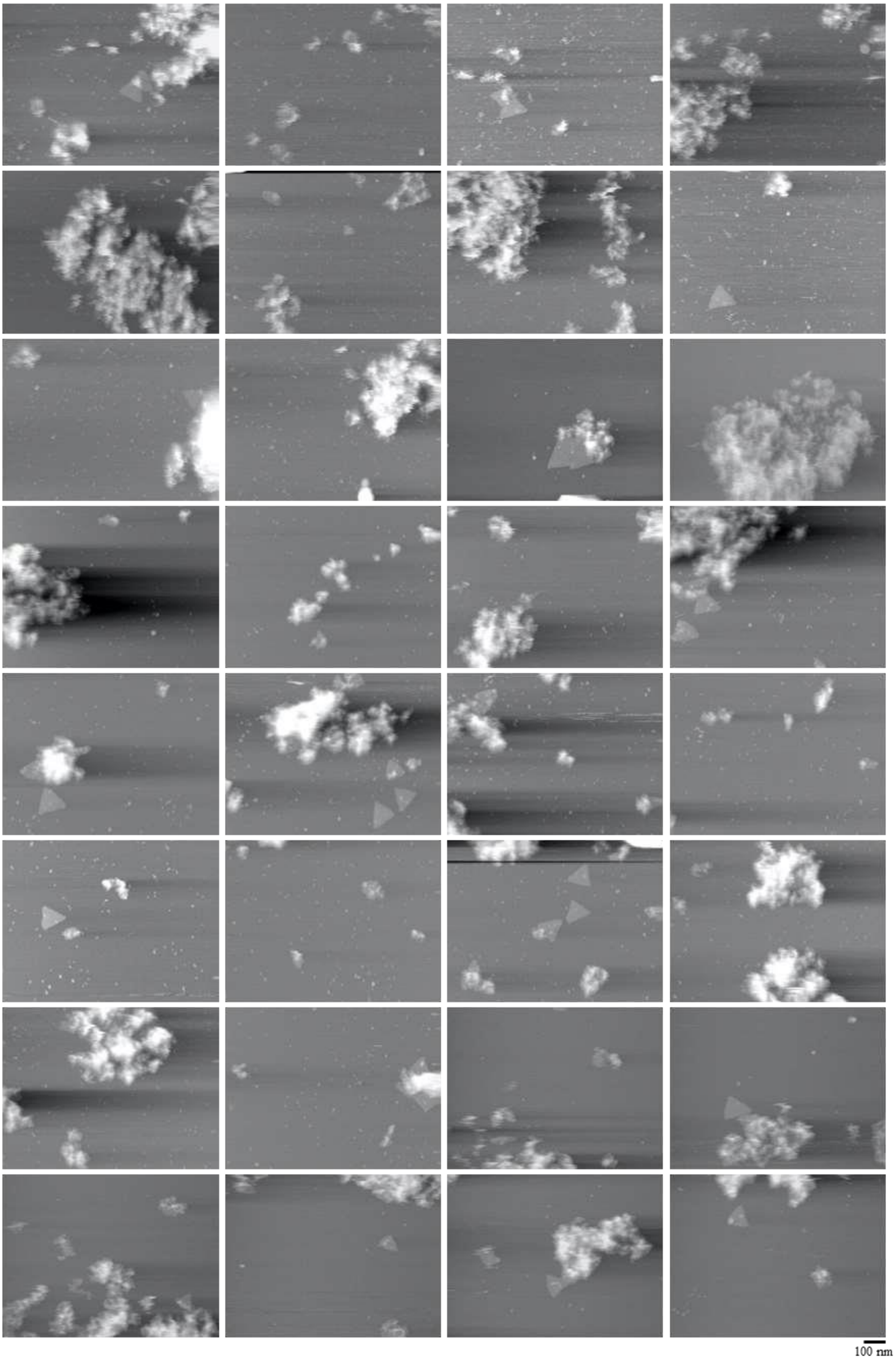
100 nm

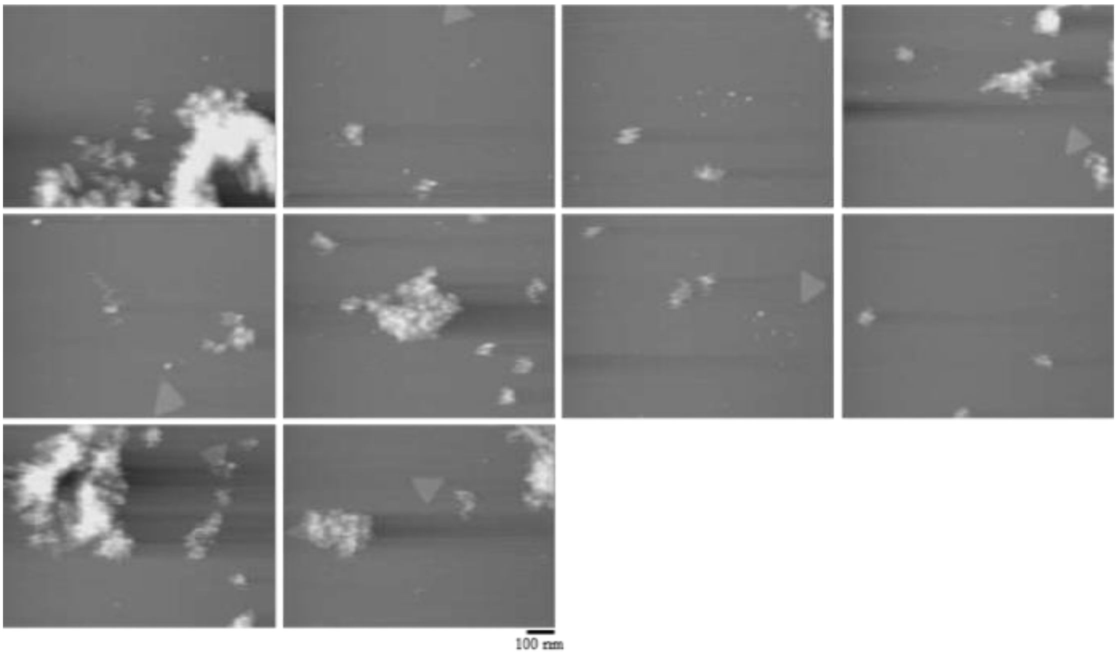

Figure S17. AFM image set of the DNA truss structures in “large”-sized droplets under "Quick_1min" annealing conditions.

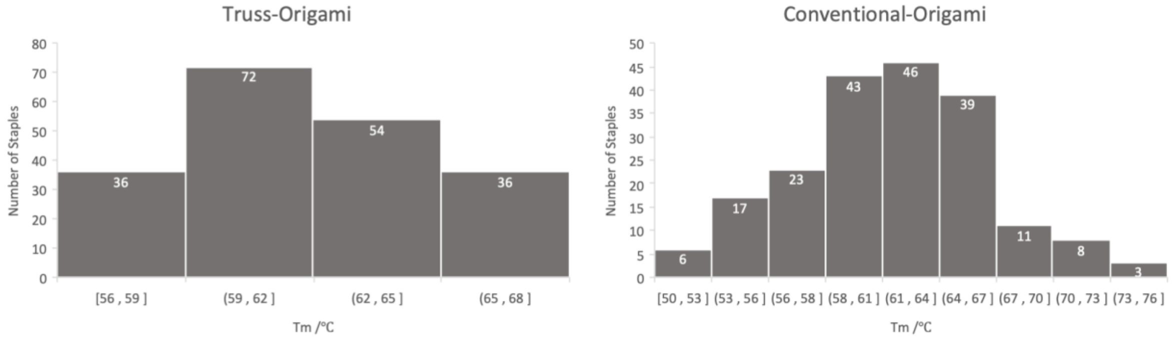

Figure S18. Distributions of the melting temperature of the staple DNA set used for the DNA origamis. The average values are: Truss-Origami: 62.2±2.8℃; Conventional-Origami: 61.8±4.8℃.

Table S1. Staple strand sequences of the used DNA origamis of the Truss structure. Their melting temperature was calculated using an online tool (http://biotools.nubic.northwestern.edu/OligoCalc.html) based on the Wallace formula (Wallace RB *et al.* (1979) Nucleic Acids Res 6:3543-3557).

staple	sequence	length	Tm/℃
Truss-S1	ATATAGAAGGCTTATCCGGTATTCTAAGAACGCGAGGCGTTTT	43 nt	66.3
Truss-S2	TCAAGATTAGTTGCTATTTCGCCCAATAGCAAGCAAATCAG	41 nt	64.6
Truss-S3	TCGTAGGAATCATTACTGCACCCAGCTACAAT	32 nt	63.1
Truss-S4	CAAGAAAAATAATATCTTTTTAGCCGTTTTTATTTTCA	38 nt	56.3
Truss-S5	CACTCATCGAGAACAAGCCCATCCTAATTTACGAGCA	37 nt	66.1
Truss-S6	TTTATCCTGAATCTTAGGTATTAAACCAAGTACCG	35 nt	60.6
Truss-S7	TATCATTCCAAGAACGAAATGAAAATAGCAGC	32 nt	59.3
Truss-S8	CAACGGAGATTTGTATATCGGCTGTCTTTCCT	32 nt	63.1
Truss-S9	TGTAGAAACCAATCAATACATGTTCAGCTAATGCAGA	37 nt	61.7
Truss-S10	AGAGGCATTTTCGAGCTTTTTAATAGATAAGTCCTGAA	38 nt	61.7
Truss-S11	ACGCGCCTGTTTATCAACCAGTAATAAGAGAATATAA	37 nt	61.7
Truss-S12	ATATAGAAGGCTTATCCGGTATTCTAAGAACGCGAGGCGTTTT	35 nt	66.3
Truss-S13	TCAAGATTAGTTGCTATTTCGCCCAATAGCAAGCAAATCAG	34 nt	64.6
Truss-S14	TCGTAGGAATCATTACTGCACCCAGCTACAAT	37 nt	63.1
Truss-S15	CAAGAAAAATAATATCTTTTTAGCCGTTTTTATTTTCA	38 nt	56.3
Truss-S16	CACTCATCGAGAACAAGCCCATCCTAATTTACGAGCA	37 nt	66.1
Truss-S17	TTTATCCTGAATCTTAGGTATTAAACCAAGTACCG	32 nt	60.6
Truss-S18	TATCATTCCAAGAACGAAATGAAAATAGCAGC	33 nt	59.3
Truss-S19	CAACGGAGATTTGTATATCGGCTGTCTTTCCT	37 nt	63.1
Truss-S20	TGTAGAAACCAATCAATACATGTTCAGCTAATGCAGA	38 nt	61.7
Truss-S21	AGAGGCATTTTCGAGCTTTTTAATAGATAAGTCCTGAA	37 nt	61.7
Truss-S22	ACGCGCCTGTTTATCAACCAGTAATAAGAGAATATAA	35 nt	61.7
Truss-S23	ATATAGAAGGCTTATCCGGTATTCTAAGAACGCGAGGCGTTTT	34 nt	66.3
Truss-S24	TCAAGATTAGTTGCTATTTCGCCCAATAGCAAGCAAATCAG	37 nt	64.6

Truss-S25	TCGTAGGAATCATTACTGCACCCAGCTACAAT	38 nt	63.1
Truss-S26	CAAGAAAAATAATATCTTTTTAGCCGTTTTTATTTTCA	37 nt	56.3
Truss-S27	CACTCATCGAGAACAAGCCCATCCTAATTTACGAGCA	32 nt	66.1
Truss-S28	TTTATCCTGAATCTTAGGTATTAAACCAAGTACCG	32 nt	60.6
Truss-S29	TATCATTCCAAGAACGAAATGAAAATAGCAGC	37 nt	59.3
Truss-S30	CAACGGAGATTTGTATATCGGCTGTCTTTCCT	38 nt	63.1
Truss-S31	TGTAGAAACCAATCAATACATGTTCAGCTAATGCAGA	37 nt	61.7
Truss-S32	AGAGGCATTTTCGAGCTTTTTAATAGATAAGTCCTGAA	35 nt	61.7
Truss-S33	ACGCGCCTGTTTATCAACCAGTAATAAGAGAATATAA	34 nt	61.7
Truss-S34	ATATAGAAGGCTTATCCGGTATTCTAAGAACGCGAGGCGTTTT	37 nt	66.3
Truss-S35	TCAAGATTAGTTGCTATTTCGCCCAATAGCAAGCAAATCAG	38 nt	64.6
Truss-S36	TCGTAGGAATCATTACTGCACCCAGCTACAAT	37 nt	63.1
Truss-S37	CAAGAAAAATAATATCTTTTTAGCCGTTTTTATTTTCA	32 nt	56.3
Truss-S38	CACTCATCGAGAACAAGCCCATCCTAATTTACGAGCA	32 nt	66.1
Truss-S39	TTTATCCTGAATCTTAGGTATTAAACCAAGTACCG	37 nt	60.6
Truss-S40	TATCATTCCAAGAACGAAATGAAAATAGCAGC	38 nt	59.3
Truss-S41	CAACGGAGATTTGTATATCGGCTGTCTTTCCT	37 nt	63.1
Truss-S42	TGTAGAAACCAATCAATACATGTTCAGCTAATGCAGA	35 nt	61.7
Truss-S43	AGAGGCATTTTCGAGCTTTTTAATAGATAAGTCCTGAA	34 nt	61.7
Truss-S44	ACGCGCCTGTTTATCAACCAGTAATAAGAGAATATAA	34 nt	61.7
Truss-S45	ATATAGAAGGCTTATCCGGTATTCTAAGAACGCGAGGCGTTTT	33 nt	66.3
Truss-S46	TCAAGATTAGTTGCTATTTCGCCCAATAGCAAGCAAATCAG	40 nt	64.6
Truss-S47	TCGTAGGAATCATTACTGCACCCAGCTACAAT	43 nt	63.1
Truss-S48	CAAGAAAAATAATATCTTTTTAGCCGTTTTTATTTTCA	40 nt	56.3
Truss-S49	CACTCATCGAGAACAAGCCCATCCTAATTTACGAGCA	37 nt	66.1
Truss-S50	TTTATCCTGAATCTTAGGTATTAAACCAAGTACCG	37 nt	60.6
Truss-S51	TATCATTCCAAGAACGAAATGAAAATAGCAGC	38 nt	59.3
Truss-S52	CAACGGAGATTTGTATATCGGCTGTCTTTCCT	34 nt	63.1
Truss-S53	TGTAGAAACCAATCAATACATGTTCAGCTAATGCAGA	35 nt	61.7
Truss-S54	AGAGGCATTTTCGAGCTTTTTAATAGATAAGTCCTGAA	37 nt	61.7
Truss-S55	ACGCGCCTGTTTATCAACCAGTAATAAGAGAATATAA	32 nt	61.7
Truss-S56	ATATAGAAGGCTTATCCGGTATTCTAAGAACGCGAGGCGTTTT	35 nt	66.3
Truss-S57	TCAAGATTAGTTGCTATTTCGCCCAATAGCAAGCAAATCAG	37 nt	64.6
Truss-S58	TCGTAGGAATCATTACTGCACCCAGCTACAAT	35 nt	63.1
Truss-S59	CAAGAAAAATAATATCTTTTTAGCCGTTTTTATTTTCA	35 nt	56.3
Truss-S60	CACTCATCGAGAACAAGCCCATCCTAATTTACGAGCA	32 nt	66.1
Truss-S61	TTTATCCTGAATCTTAGGTATTAAACCAAGTACCG	36 nt	60.6
Truss-S62	TATCATTCCAAGAACGAAATGAAAATAGCAGC	38 nt	59.3
Truss-S63	CAACGGAGATTTGTATATCGGCTGTCTTTCCT	35 nt	63.1
Truss-S64	TGTAGAAACCAATCAATACATGTTCAGCTAATGCAGA	37 nt	61.7
Truss-S65	AGAGGCATTTTCGAGCTTTTTAATAGATAAGTCCTGAA	35 nt	61.7
Truss-S66	ACGCGCCTGTTTATCAACCAGTAATAAGAGAATATAA	35 nt	61.7
Truss-S67	ATATAGAAGGCTTATCCGGTATTCTAAGAACGCGAGGCGTTTT	35 nt	66.3

Truss-S68	TCAAGATTAGTTGCTATTTCGCCCAATAGCAAGCAAATCAG	37 nt	64.6
Truss-S69	TCGTAGGAATCATTACTGCACCCAGCTACAAT	37 nt	63.1
Truss-S70	CAAGAAAAATAATATCTTTTTAGCCGTTTTTATTTTCA	35 nt	56.3
Truss-S71	CACTCATCGAGAACAAGCCCATCCTAATTTACGAGCA	34 nt	66.1
Truss-S72	TTTATCCTGAATCTTAGGTATTAAACCAAGTACCG	37 nt	60.6
Truss-S73	TATCATTCCAAGAACGAAATGAAAATAGCAGC	37 nt	59.3
Truss-S74	CAACGGAGATTTGTATATCGGCTGTCTTTCCT	32 nt	63.1
Truss-S75	TGTAGAAACCAATCAATACATGTTCAGCTAATGCAGA	34 nt	61.7
Truss-S76	AGAGGCATTTTCGAGCTTTTTAATAGATAAGTCCTGAA	35 nt	61.7
Truss-S77	ACGCGCCTGTTTATCAACCAGTAATAAGAGAATATAA	34 nt	61.7
Truss-S78	ATATAGAAGGCTTATCCGGTATTCTAAGAACGCGAGGCGTTTT	35 nt	66.3
Truss-S79	TCAAGATTAGTTGCTATTTCGCCCAATAGCAAGCAAATCAG	34 nt	64.6
Truss-S80	TCGTAGGAATCATTACTGCACCCAGCTACAAT	37 nt	63.1
Truss-S81	CAAGAAAAATAATATCTTTTTAGCCGTTTTTATTTTCA	36 nt	56.3
Truss-S82	CACTCATCGAGAACAAGCCCATCCTAATTTACGAGCA	35 nt	66.1
Truss-S83	TTTATCCTGAATCTTAGGTATTAAACCAAGTACCG	32 nt	60.6
Truss-S84	TATCATTCCAAGAACGAAATGAAAATAGCAGC	36 nt	59.3
Truss-S85	CAACGGAGATTTGTATATCGGCTGTCTTTCCT	38 nt	63.1
Truss-S86	TGTAGAAACCAATCAATACATGTTCAGCTAATGCAGA	35 nt	61.7
Truss-S87	AGAGGCATTTTCGAGCTTTTTAATAGATAAGTCCTGAA	38 nt	61.7
Truss-S88	ACGCGCCTGTTTATCAACCAGTAATAAGAGAATATAA	35 nt	61.7
Truss-S89	ATATAGAAGGCTTATCCGGTATTCTAAGAACGCGAGGCGTTTT	35 nt	66.3
Truss-S90	TCAAGATTAGTTGCTATTTCGCCCAATAGCAAGCAAATCAG	34 nt	64.6
Truss-S91	TCGTAGGAATCATTACTGCACCCAGCTACAAT	37 nt	63.1
Truss-S92	CAAGAAAAATAATATCTTTTTAGCCGTTTTTATTTTCA	37 nt	56.3
Truss-S93	CACTCATCGAGAACAAGCCCATCCTAATTTACGAGCA	35 nt	66.1
Truss-S94	TTTATCCTGAATCTTAGGTATTAAACCAAGTACCG	34 nt	60.6
Truss-S95	TATCATTCCAAGAACGAAATGAAAATAGCAGC	37 nt	59.3
Truss-S96	CAACGGAGATTTGTATATCGGCTGTCTTTCCT	37 nt	63.1
Truss-S97	TGTAGAAACCAATCAATACATGTTCAGCTAATGCAGA	35 nt	61.7
Truss-S98	AGAGGCATTTTCGAGCTTTTTAATAGATAAGTCCTGAA	34 nt	61.7
Truss-S99	ACGCGCCTGTTTATCAACCAGTAATAAGAGAATATAA	37 nt	61.7
Truss-S100	ATATAGAAGGCTTATCCGGTATTCTAAGAACGCGAGGCGTTTT	37 nt	66.3
Truss-S101	TCAAGATTAGTTGCTATTTCGCCCAATAGCAAGCAAATCAG	35 nt	64.6
Truss-S102	TCGTAGGAATCATTACTGCACCCAGCTACAAT	34 nt	63.1
Truss-S103	CAAGAAAAATAATATCTTTTTAGCCGTTTTTATTTTCA	37 nt	56.3
Truss-S104	CACTCATCGAGAACAAGCCCATCCTAATTTACGAGCA	37 nt	66.1
Truss-S105	TTTATCCTGAATCTTAGGTATTAAACCAAGTACCG	32 nt	60.6
Truss-S106	TATCATTCCAAGAACGAAATGAAAATAGCAGC	34 nt	59.3
Truss-S107	CAACGGAGATTTGTATATCGGCTGTCTTTCCT	35 nt	63.1
Truss-S108	TGTAGAAACCAATCAATACATGTTCAGCTAATGCAGA	34 nt	61.7
Truss-S109	AGAGGCATTTTCGAGCTTTTTAATAGATAAGTCCTGAA	35 nt	61.7
Truss-S110	ACGCGCCTGTTTATCAACCAGTAATAAGAGAATATAA	34 nt	61.7

Truss-S111	ATATAGAAGGCTTATCCGGTATTCTAAGAACGCGAGGCGTTTT	35 nt	66.3
Truss-S112	TCAAGATTAGTTGCTATTTCGCCCAATAGCAAGCAAATCAG	34 nt	64.6
Truss-S113	TCGTAGGAATCATTACTGCACCCAGCTACAAT	35 nt	63.1
Truss-S114	CAAGAAAAATAATATCTTTTTAGCCGTTTTTATTTTCA	35 nt	56.3
Truss-S115	CACTCATCGAGAACAAGCCCATCCTAATTTACGAGCA	37 nt	66.1
Truss-S116	TTTATCCTGAATCTTAGGTATTAAACCAAGTACCG	35 nt	60.6
Truss-S117	TATCATTCCAAGAACGAAATGAAAATAGCAGC	35 nt	59.3
Truss-S118	CAACGGAGATTTGTATATCGGCTGTCTTTCCT	32 nt	63.1
Truss-S119	TGTAGAAACCAATCAATACATGTTCAGCTAATGCAGA	36 nt	61.7
Truss-S120	AGAGGCATTTTCGAGCTTTTTAATAGATAAGTCCTGAA	38 nt	61.7
Truss-S121	ACGCGCCTGTTTATCAACCAGTAATAAGAGAATATAA	35 nt	61.7
Truss-S122	ATATAGAAGGCTTATCCGGTATTCTAAGAACGCGAGGCGTTTT	37 nt	66.3
Truss-S123	TCAAGATTAGTTGCTATTTCGCCCAATAGCAAGCAAATCAG	35 nt	64.6
Truss-S124	TCGTAGGAATCATTACTGCACCCAGCTACAAT	35 nt	63.1
Truss-S125	CAAGAAAAATAATATCTTTTTAGCCGTTTTTATTTTCA	35 nt	56.3
Truss-S126	CACTCATCGAGAACAAGCCCATCCTAATTTACGAGCA	37 nt	66.1
Truss-S127	TTTATCCTGAATCTTAGGTATTAAACCAAGTACCG	37 nt	60.6
Truss-S128	TATCATTCCAAGAACGAAATGAAAATAGCAGC	35 nt	59.3
Truss-S129	CAACGGAGATTTGTATATCGGCTGTCTTTCCT	34 nt	63.1
Truss-S130	TGTAGAAACCAATCAATACATGTTCAGCTAATGCAGA	37 nt	61.7
Truss-S131	AGAGGCATTTTCGAGCTTTTTAATAGATAAGTCCTGAA	37 nt	61.7
Truss-S132	ACGCGCCTGTTTATCAACCAGTAATAAGAGAATATAA	35 nt	61.7
Truss-S133	ATATAGAAGGCTTATCCGGTATTCTAAGAACGCGAGGCGTTTT	34 nt	66.3
Truss-S134	TCAAGATTAGTTGCTATTTCGCCCAATAGCAAGCAAATCAG	37 nt	64.6
Truss-S135	TCGTAGGAATCATTACTGCACCCAGCTACAAT	37 nt	63.1
Truss-S136	CAAGAAAAATAATATCTTTTTAGCCGTTTTTATTTTCA	35 nt	56.3
Truss-S137	CACTCATCGAGAACAAGCCCATCCTAATTTACGAGCA	34 nt	66.1
Truss-S138	TTTATCCTGAATCTTAGGTATTAAACCAAGTACCG	37 nt	60.6
Truss-S139	TATCATTCCAAGAACGAAATGAAAATAGCAGC	37 nt	59.3
Truss-S140	CAACGGAGATTTGTATATCGGCTGTCTTTCCT	35 nt	63.1
Truss-S141	TGTAGAAACCAATCAATACATGTTCAGCTAATGCAGA	34 nt	61.7
Truss-S142	AGAGGCATTTTCGAGCTTTTTAATAGATAAGTCCTGAA	37 nt	61.7
Truss-S143	ACGCGCCTGTTTATCAACCAGTAATAAGAGAATATAA	37 nt	61.7
Truss-S144	ATATAGAAGGCTTATCCGGTATTCTAAGAACGCGAGGCGTTTT	35 nt	66.3
Truss-S145	TCAAGATTAGTTGCTATTTCGCCCAATAGCAAGCAAATCAG	34 nt	64.6
Truss-S146	TCGTAGGAATCATTACTGCACCCAGCTACAAT	37 nt	63.1
Truss-S147	CAAGAAAAATAATATCTTTTTAGCCGTTTTTATTTTCA	37 nt	56.3
Truss-S148	CACTCATCGAGAACAAGCCCATCCTAATTTACGAGCA	32 nt	66.1
Truss-S149	TTTATCCTGAATCTTAGGTATTAAACCAAGTACCG	34 nt	60.6
Truss-S150	TATCATTCCAAGAACGAAATGAAAATAGCAGC	35 nt	59.3
Truss-S151	CAACGGAGATTTGTATATCGGCTGTCTTTCCT	34 nt	63.1
Truss-S152	TGTAGAAACCAATCAATACATGTTCAGCTAATGCAGA	35 nt	61.7
Truss-S153	AGAGGCATTTTCGAGCTTTTTAATAGATAAGTCCTGAA	34 nt	61.7

Truss-S154	ACGCGCCTGTTTATCAACCAGTAATAAGAGAATATAA	35 nt	61.7
Truss-S155	ATATAGAAGGCTTATCCGGTATTCTAAGAACGCGAGGCGTTTT	34 nt	66.3
Truss-S156	TCAAGATTAGTTGCTATTTCGCCCAATAGCAAGCAAATCAG	35 nt	64.6
Truss-S157	TCGTAGGAATCATTACTGCACCCAGCTACAAT	34 nt	63.1
Truss-S158	CAAGAAAAATAATATCTTTTTAGCCGTTTTTATTTTCA	35 nt	56.3
Truss-S159	CACTCATCGAGAACAAGCCCATCCTAATTTACGAGCA	34 nt	66.1
Truss-S160	TTTATCCTGAATCTTAGGTATTAAACCAAGTACCG	35 nt	60.6
Truss-S161	TATCATTCCAAGAACGAAATGAAAATAGCAGC	35 nt	59.3
Truss-S162	CAACGGAGATTTGTATATCGGCTGTCTTTCCT	37 nt	63.1
Truss-S163	TGTAGAAACCAATCAATACATGTTCAGCTAATGCAGA	36 nt	61.7
Truss-S164	AGAGGCATTTTCGAGCTTTTTAATAGATAAGTCCTGAA	33 nt	61.7
Truss-S165	ACGCGCCTGTTTATCAACCAGTAATAAGAGAATATAA	35 nt	61.7
Truss-S166	ATATAGAAGGCTTATCCGGTATTCTAAGAACGCGAGGCGTTTT	36 nt	66.3
Truss-S167	TCAAGATTAGTTGCTATTTCGCCCAATAGCAAGCAAATCAG	37 nt	64.6
Truss-S168	TCGTAGGAATCATTACTGCACCCAGCTACAAT	40 nt	63.1
Truss-S169	CAAGAAAAATAATATCTTTTTAGCCGTTTTTATTTTCA	43 nt	56.3
Truss-S170	CACTCATCGAGAACAAGCCCATCCTAATTTACGAGCA	40 nt	66.1
Truss-S171	TTTATCCTGAATCTTAGGTATTAAACCAAGTACCG	37 nt	60.6
Truss-S172	TATCATTCCAAGAACGAAATGAAAATAGCAGC	37 nt	59.3
Truss-S173	CAACGGAGATTTGTATATCGGCTGTCTTTCCT	38 nt	63.1
Truss-S174	TGTAGAAACCAATCAATACATGTTCAGCTAATGCAGA	37 nt	61.7
Truss-S175	AGAGGCATTTTCGAGCTTTTTAATAGATAAGTCCTGAA	37 nt	61.7
Truss-S176	ACGCGCCTGTTTATCAACCAGTAATAAGAGAATATAA	38 nt	61.7
Truss-S177	ATATAGAAGGCTTATCCGGTATTCTAAGAACGCGAGGCGTTTT	37 nt	66.3
Truss-S178	TCAAGATTAGTTGCTATTTCGCCCAATAGCAAGCAAATCAG	37 nt	64.6
Truss-S179	TCGTAGGAATCATTACTGCACCCAGCTACAAT	38 nt	63.1
Truss-S180	CAAGAAAAATAATATCTTTTTAGCCGTTTTTATTTTCA	37 nt	56.3
Truss-S181	CACTCATCGAGAACAAGCCCATCCTAATTTACGAGCA	37 nt	66.1
Truss-S182	TTTATCCTGAATCTTAGGTATTAAACCAAGTACCG	38 nt	60.6
Truss-S183	TATCATTCCAAGAACGAAATGAAAATAGCAGC	37 nt	59.3
Truss-S184	CAACGGAGATTTGTATATCGGCTGTCTTTCCT	37 nt	63.1
Truss-S185	TGTAGAAACCAATCAATACATGTTCAGCTAATGCAGA	38 nt	61.7
Truss-S186	AGAGGCATTTTCGAGCTTTTTAATAGATAAGTCCTGAA	37 nt	61.7
Truss-S187	ACGCGCCTGTTTATCAACCAGTAATAAGAGAATATAA	37 nt	61.7
Truss-S188	ATATAGAAGGCTTATCCGGTATTCTAAGAACGCGAGGCGTTTT	38 nt	66.3
Truss-S189	TCAAGATTAGTTGCTATTTCGCCCAATAGCAAGCAAATCAG	37 nt	64.6
Truss-S190	TCGTAGGAATCATTACTGCACCCAGCTACAAT	37 nt	63.1
Truss-S191	CAAGAAAAATAATATCTTTTTAGCCGTTTTTATTTTCA	38 nt	56.3
Truss-S192	CACTCATCGAGAACAAGCCCATCCTAATTTACGAGCA	37 nt	66.1
Truss-S193	TTTATCCTGAATCTTAGGTATTAAACCAAGTACCG	38 nt	60.6
Truss-S194	TATCATTCCAAGAACGAAATGAAAATAGCAGC	38 nt	59.3
Truss-S195	CAACGGAGATTTGTATATCGGCTGTCTTTCCT	37 nt	63.1
Truss-S196	TGTAGAAACCAATCAATACATGTTCAGCTAATGCAGA	37 nt	61.7

Truss-S197	AGAGGCATTTTCGAGCTTTTTAATAGATAAGTCCTGAA	40 nt	61.7
Truss-S198	ACGCGCCTGTTTATCAACCAGTAATAAGAGAATATAA	37 nt	61.7

Table S2. Staple strand sequences of the used DNA origami of the conventional structure.

staple	sequence	length	Tm/℃
Origami-S1	ATACCTAACAATATTACCGCCA	22 nt	53.0
Origami-S2	TAATTCGAAAAAAGATTAAGAGG	23 nt	49.8
Origami-S3	CAGGAGGCCGATTAAAGGGATTTT	24 nt	58.8
Origami-S4	GCCCAATAAATCAAGATTAGTTGC	24 nt	55.3
Origami-S5	AGCCATATTATTTATCGACGGGAG	24 nt	57.1
Origami-S6	TATGGGATCCAAAAAAAAGGCTCC	24 nt	57.1
Origami-S7	TAAAACGAAAGAGGCATGTGTCGA	24 nt	57.1
Origami-S8	AAACCCTGCAAATGAAAAATCTACCACCAG	30 nt	60.5
Origami-S9	TAATACAATCTTTAGGAGCACTCAAATATC	30 nt	56.4
Origami-S10	AAGCCCAAGCTATCTTACCGAAGCCCTTTT	30 nt	63.3
Origami-S11	TCACGACGGTGCTGCAAGGCGATCCATTCA	30 nt	67.4
Origami-S12	CAAAATTCAATTACCTGAGCAATCGGGAGA	30 nt	60.5
Origami-S13	ATTAACCGGCCGCCACCAGAACCGCCACCA	30 nt	70.1
Origami-S14	CATCTGCCATGGGATAGGTCACGCCAGCTT	30 nt	67.4
Origami-S15	GCAACAGCCGTATTGGGCGCCAGACTGCCC	30 nt	71.5
Origami-S16	CCCTCGTGTCCAATACTGCGGAATGCTTTA	30 nt	64.6
Origami-S17	AATTGCTGGCTCCTTTTGATAAGCGCGTTT	30 nt	61.9
Origami-S18	AGGAGGTATGTACCGTAACACTGTAGCATT	30 nt	61.9
Origami-S19	TTAGAACATATAATCCTGATTGATTTTGCG	30 nt	56.4
Origami-S20	AACAATAAACGTCAGATGAATATGGAAGGG	30 nt	59.2
Origami-S21	TTTTCCCTGTGAGTGAATAACCCAAGAAAA	30 nt	59.2
Origami-S22	GGCTGCGCCGCTTCTGGTGCCGGAACCGTG	30 nt	74.2
Origami-S23	GAAGCCTTCGGTTGTACCAAAAATTAACAT	30 nt	59.2
Origami-S24	TAGGTTGTATCAAAATCATAGGTTAATTAA	30 nt	53.7
Origami-S25	AATTACCTAACGAGCGTCTTTCCAATCTTA	30 nt	59.2
Origami-S26	AGTTTGACTTCCATATAACAGTTAGAGCTT	30 nt	57.8
Origami-S27	GCTTTCCAAATGAGTGAGCTAACCCTGTGT	30 nt	63.3
Origami-S28	CCACAGACAACAGTTTCAGCGGGCGAATAA	30 nt	64.6
Origami-S29	AATGGTTTTTTTCAAATATATTCTCCGGCT	30 nt	56.4
Origami-S30	TAGCTTAGTACAATTTTATCCTGTATTTTG	30 nt	55.1
Origami-S31	AACGGGTATAGAAGGCTTATCCGCTCCCGA	30 nt	66.0
Origami-S32	GAAGAAAAGATACATAACGCCATATCATAA	30 nt	56.4
Origami-S33	GTAAAACAAGTCAGAGGGTAATTGAACACC	30 nt	60.5
Origami-S34	TGATGCAATTCATCGTAGGAATCCAAGCCG	30 nt	63.3
Origami-S35	GAAATTGTACCGAGCTCGAATTCTTCCCAG	30 nt	63.3
Origami-S36	AATCAACACTTATTACGCAGTATGCATGAT	30 nt	57.8
Origami-S37	AAAATCACCAGTAGCACCATTACCCATCGA	30 nt	61.9
Origami-S38	ATCATCATGGAAACCGAGGAAACACAAAGT	30 nt	60.5

Origami-S39	TACCAAGTAGTTACAAAATAAACAGAGCCT	30 nt	57.8
Origami-S40	CTCCCTCAGTTGAGGCAGGTCAGTCCTCAT	30 nt	67.4
Origami-S41	AATATTTTACGGAAATTATTCATGGAAGGT	30 nt	55.1
Origami-S42	TTGTTTAGAGAATAACATAAAAATTGAGTT	30 nt	52.3
Origami-S43	TAGCAGCATTGCCATCTTTTCATGAACCGC	30 nt	63.3
Origami-S44	TTTACAAATACCCAAAAGAACTGGCAATAA	30 nt	56.4
Origami-S45	GTAAACGTAAAGCCCCAAAAACACTGGAGC	30 nt	63.3
Origami-S46	TCGGAACTGCCGTCGAGAGGGTCCGTACTC	30 nt	70.1
Origami-S47	TCATCAACCGCCATCAAAAATAATTAAATT	30 nt	55.1
Origami-S48	CCAATAAATGAAAAGGTGGCATCTAGATTT	30 nt	57.8
Origami-S49	ACCTGAAAGTAATAAAAGGGACTCATGGAA	30 nt	59.2
Origami-S50	CAGAAGAATAGCCCTAAAACATCCCTTCTG	30 nt	61.9
Origami-S51	AAACAAGAGAGATCTACAAAGGCAGACAGT	30 nt	60.5
Origami-S52	AGGCATTTTCGAGCCAGTAATAAATTCTGT	30 nt	59.2
Origami-S53	AGTGTTTTCATGGCTTTTGATGACCAGTAA	30 nt	59.2
Origami-S54	CAAATCACTGTGTAGGTAAAGATTGCGGGA	30 nt	61.9
Origami-S55	AAGGCGTTAACAATAGATAAGTCACGCGCC	30 nt	63.3
Origami-S56	ATTAACACAACCGATTGAGGGAGAAAAGGG	30 nt	61.9
Origami-S57	CCAGACGATACGAGCATGTAGAATTCCAAG	30 nt	63.3
Origami-S58	TAAAGCCAGGTCAGTGCCTTGAGTTTGACG	30 nt	64.6
Origami-S59	CTTTGACCCAACGGAGATTTGTAGACCAAC	30 nt	63.3
Origami-S60	ATGGATTAACCGTCACCGACTTGTAAAGGT	30 nt	61.9
Origami-S61	GAACAAAGAACGTTATTAATTTTCAATAGA	30 nt	53.7
Origami-S62	CATAAAGGTGGCAACATATAAAATTGTCAC	30 nt	57.8
Origami-S63	AGAAGAACTAGCGCGTTTTCATCTTTAGCG	30 nt	61.9
Origami-S64	CTTTCCACGGAAGCATAAAGTGTCACACAAC	31 nt	63.2
Origami-S65	AACAGTTCAGAAAACGAGAATGAGTTTAGAC	31 nt	59.2
Origami-S66	AATCATTGTGAATTACCTTATGCGACGTTGG	31 nt	60.5
Origami-S67	CCGATAGGGAAGATCGCACTCCAGACAGTAT	31 nt	65.8
Origami-S68	AAATTTTTCATTAAACGGGTAAATCATGAGG	31 nt	56.6
Origami-S69	GCTTTGATCTCCGTGGGAACAAATAACAACC	31 nt	63.2
Origami-S70	GTGAATAAAGCAATAAAGCCTCAAAGAATTA	31 nt	56.6
Origami-S71	CATGTTAAAATTAATGCCGGAGAAACCGTTC	31 nt	60.5
Origami-S72	ACCCTGACTATTATAGTCAGAAGCAAAGCGG	31 nt	63.2
Origami-S73	TTCGAGGTTCGCTATTACGCCAGGATCGGTG	31 nt	67.2
Origami-S74	CGTCGGATGGACTAAAGACTTTTCTACAGAG	31 nt	63.2
Origami-S75	TTTTAAATGAAAGATTCATCAGTACATTATT	31 nt	52.6
Origami-S76	GCTTGCATTTGTATCGGTTTATCAAAAGGAG	31 nt	60.5
Origami-S77	TGAGAGAGAGAAGGATTAGGATTCTGAGACT	31 nt	61.9
Origami-S78	CAGCAGCGAAAGACAGCATCGGACGCATAAC	31 nt	67.2
Origami-S79	CCAAATCCCTCATATATTTTAAATAAAAATT	31 nt	52.6
Origami-S80	CCTCAAGTTGCAGCAAGCGGTCCCCTGGCCC	31 nt	72.4
Origami-S81	GCAAACTCGAAGTTTTGCCAGAGGAGGCTTT	31 nt	64.5

Origami-S82	TTTGAAAGTAGGCTGGCTGACCTTCATCAAG	31 nt	63.2
Origami-S83	ACTAGCATACGAAGGCACCAACCATACGTAA	31 nt	63.2
Origami-S84	CAGAACGTGTTTAGCTATATTTTCAAATGGT	31 nt	57.9
Origami-S85	TGCAAAACAACAGGTCAGGATTAGACCGGAA	31 nt	63.2
Origami-S86	GCATTAATAGAGCCACCACCCTCAGAACCGC	31 nt	67.2
Origami-S87	TAATTTTTTCACGTTGAAAATCTTTTGCTAA	31 nt	53.9
Origami-S88	CGTGGACTCCAACGTCAAAGGGCTGCCTATT	31 nt	67.2
Origami-S89	CGATATATTCGGTCGCTGAGGCTGTCACCCT	31 nt	67.2
Origami-S90	CGGGCCTCTGAATTTCTTAAACAAGCTTGCT	31 nt	63.2
Origami-S91	CGGCCTCATTGCGCCGACAATGAGCTTGATA	31 nt	67.2
Origami-S92	TGTACTGGTTAGAATCAGAGCGGGAGCTAAA	31 nt	63.2
Origami-S93	TAGCTGATCTTAGCCGGAACGAGACCTGCTC	31 nt	67.2
Origami-S94	AAGTTTCGTTAAATCAGCTCATTTTCGCATT	31 nt	57.9
Origami-S95	CACCCTCGAATCGGCCAACGCGCTGCCAGCT	31 nt	72.4
Origami-S96	CAATAACCAGTAGTAAATTGGGCAGAAACAC	31 nt	60.5
Origami-S97	ACAGGTAATGCAACTAAAGTACGTCAACATG	31 nt	60.5
Origami-S98	CCTTTAAGCCTGCAGGTCGACTCAGTGCCAA	31 nt	67.2
Origami-S99	TTTAGAACAACGTAACAAAGCTGTTCATTAC	31 nt	57.9
Origami-S100	AATTGAGAATCGCCATATTTAACAACGCCAA	31 nt	59.2
Origami-S101	ATACGAGCGACGTTAGTAAATGATTTGTCGT	31 nt	60.5
Origami-S102	GGGTTGAGTGTTGTTCCAGTTTGGAACAAGA	31 nt	63.2
Origami-S103	TGCCACTGTCAATCATATGTACCATCGTAAA	31 nt	60.5
Origami-S104	GCAAAATTAGGCTTGCCCTGACGCTCATTCA	31 nt	64.5
Origami-S105	TGATAAATAAAGAATACACTAAAACACTCAT	31 nt	53.9
Origami-S106	AGGAACCCTTAGTACCGCCACCCTGTACCAGG	32 nt	69.5
Origami-S107	CTGAGTAACATCAATATGATATTCGGGTAGCT	32 nt	60.6
Origami-S108	TTTTTATTATCCAATCGCAAGACAGTAAATGC	32 nt	58.0
Origami-S109	AGGGGGATTTGTAAAACGACGGCCTAGAGGAT	32 nt	65.7
Origami-S110	TTTAACGGGAATGGAAAGCGCAGTCCAGCATT	32 nt	64.4
Origami-S111	TGAACGGTTAGCAGCCTTTACAGAACGTCAAA	32 nt	63.1
Origami-S112	TCACCGGAACAAACTACAACGCCTGAGTTTCG	32 nt	65.7
Origami-S113	CGGTCATAAACCGCCACCCTCAGAACCACCAG	32 nt	69.5
Origami-S114	GCGCATTACCAATCCAAATAAGAAACGATTTT	32 nt	59.3
Origami-S115	GTCTATCACGCGTACTATGGTTGCTAACAGTG	32 nt	64.4
Origami-S116	AATAGGAAATTAAATGTGAGCGAGCGGCGGAT	32 nt	63.1
Origami-S117	CCCCGGGTTATCCGCTCACAATTCAAAGCCTG	32 nt	68.2
Origami-S118	AGCTAAATTATTTCAACGCAAGGATGCAATGC	32 nt	60.6
Origami-S119	TAATCAGATAATATTTTGTTAAAATTTTAACC	32 nt	51.6
Origami-S120	CCTTGATACGGAATAGGTGTATCATGATATAA	32 nt	59.3
Origami-S121	GGGTGCCTGTCGGGAAACCTGTCGGGGGAGAG	32 nt	73.4
Origami-S122	TAAGAAAAGAGATAACCCACAAGAACAGGGAA	32 nt	60.6
Origami-S123	GCGCGAGCTCATACAGGCAAGGCAGAGCATAA	32 nt	68.2
Origami-S124	ATTTTTGAGAATCGATGAACGGTACCGGTTGA	32 nt	61.8

Origami-S125	CTTTAATTAATATAATGCTGTAGCGTGTCTGG	32 nt	59.3
Origami-S126	AGCCAGCACAATCAATATCTGGTCGAAGGTTA	32 nt	63.1
Origami-S127	TCTAAAATTTTGAGGATTTAGAAGATTAAATC	32 nt	54.1
Origami-S128	GTTAGCAAACGTAGAATACCAGCGCCAAAGAC	32 nt	64.4
Origami-S129	AAAGTACCATTGAATCCCCCTCAAATCGTCAT	32 nt	61.8
Origami-S130	AAATATTGTGAATGGCTATTAGTCGCACAGAC	32 nt	60.6
Origami-S131	TAGAAAGGAACGTCACCAATGAAACATTAGCA	32 nt	60.6
Origami-S132	GAAAAATAACTCATCGAGAACAAGATTACCGC	32 nt	60.6
Origami-S133	ATTCATTTAATTACATTTAACAATTACATAAA	32 nt	50.3
Origami-S134	TGTTTATCAAATAAGAATAAACACTGATAAAT	32 nt	52.9
Origami-S135	TCACCAGTACCAGAGCCACCACCGAATCAAAA	32 nt	65.7
Origami-S136	TACCAGAAATTCCTGATTATCAGAGCGGAATT	32 nt	60.6
Origami-S137	TCAATATATTAGAATCCTTGAAAAGAGTCAAT	32 nt	55.4
Origami-S138	TTTGCTCACAGAACCGCCACCCTCATTTTCAG	32 nt	65.7
Origami-S139	GGATAGCACGTAACGATCTAAAGTATTTTCTG	32 nt	60.6
Origami-S140	CTGAACAAGAAATAAAGAAATTGCATTTGCAC	32 nt	58.0
Origami-S141	TCAGACTGTCAAACTATCGGCCTTGCCTGAGT	32 nt	65.7
Origami-S142	AGTAAAATCCATAAATCAAAAATCAGGTCTTT	32 nt	55.4
Origami-S143	CAACAACCATCGCCCAACGAGGGTAGCAACGG	32 nt	69.5
Origami-S144	GACAGGAGGAGCCGCCACCCTCAGGCCCCCTT	32 nt	74.6
Origami-S145	ATAATCGGATAGTAAGAGCAACACAAAGGAAT	32 nt	59.3
Origami-S146	CCCTCAGATTGTAGCAATACTTCTTCACGCAA	32 nt	63.1
Origami-S147	TAAGACTCGTTGAAAGGAATTGAGAGTTGGCA	32 nt	61.8
Origami-S148	GCGTCATATATAATCAGTGAGGCCTCCTGAGA	32 nt	64.4
Origami-S149	ACTAATGCAATCTACGTTAATAAATCAACTTT	32 nt	55.4
Origami-S150	CTTGCGGGAGGTTTTGAAGCCTTAGCAAGCAA	32 nt	65.7
Origami-S151	CTTTGCCCGAAACCACCAGAAGGATGATGGCA	32 nt	67.0
Origami-S152	ATTCATCACTACCATATCAAAATTGTAGATTT	32 nt	55.4
Origami-S153	AGTAATCTATAAGGGAACCGAACTTCATCGCC	32 nt	63.1
Origami-S154	AATTTGCCTACAAAATCGCGCAGAGCTTTGAA	32 nt	61.8
Origami-S155	ATCAGATATTAAACCAAGTACCGCATATCCCA	32 nt	60.6
Origami-S156	GTATAGCCTTCACAAACAAATAAAACGATTGG	32 nt	59.3
Origami-S157	TAATTACTAGCCAACGCTCAACAGTAGGGCTT	32 nt	63.1
Origami-S158	AAGTTTCACATTAGATACATTTCGCATTTGGG	32 nt	59.3
Origami-S159	TCAGGTTTACGGATTCGCCTGATTGGCGAATT	32 nt	64.4
Origami-S160	GAATTATCTTTACATTGGCAGATTCGTCTGAA	32 nt	59.3
Origami-S161	ATTGCATCGCTTCAAAGCGAACCAGAGAGTAC	32 nt	64.4
Origami-S162	AGGCCGGAAACAACTAAAGGAATTAGTGAGAA	32 nt	61.8
Origami-S163	TTGCCCCAAAATCAAAAGAATAGCCCGAGATA	32 nt	61.8
Origami-S164	AGCCGCCGCTCTGAATTTACCGTTTACAGGAG	32 nt	67.0
Origami-S165	ATTAGCGTCCGTAATCAGTAGCGAGAGCCAGC	32 nt	67.0
Origami-S166	CATGTAATGTTCAGCTAATGCAGACTGAACAA	32 nt	60.6
Origami-S167	CCCGTATAAACAGTTAATGCCCCCGAAAAACC	32 nt	64.4

Origami-S168	CCAACGCTTTTTTAATGGAAACAGTTCATTTG	32 nt	59.3
Origami-S169	TGACCGTAAGTTTGAGGGGACGACGCCAGCTT	32 nt	68.2
Origami-S170	AAATATTCGACAAAAGGTAAAGTAGAGAATAT	32 nt	55.4
Origami-S171	CACCCAGCATTAAGACGCTGAGAACATAGCGA	32 nt	65.7
Origami-S172	CGGATAAGCTATTATTCTGAAACATTAAAGAA	32 nt	56.7
Origami-S173	TCCGGCACAACTGTTGGGAAGGGCCTGGCGAA	32 nt	70.8
Origami-S174	GCGGTTTGTGATTGCCCTTCACCGACGCTGGT	32 nt	69.5
Origami-S175	TCCTAATTCGACAATAAACAACATTTAGGCAG	32 nt	59.3
Origami-S176	GCGAACTGTAAAACAGAGGTGAGGACGCTGAG	32 nt	67.0
Origami-S177	AATGAAAAGTACAGACCAGGCGCAAGGACAGA	32 nt	64.4
Origami-S178	AATCAATAGAAAATTCATATGGTTAATACATA	32 nt	52.9
Origami-S179	ACACGACCAGCGTAAGAATACGTGTTTAATGC	32 nt	63.1
Origami-S180	AGCACGTATAACGTGCTTTCCTCGTAATAAGT	32 nt	61.8
Origami-S181	TAACGGAACAATTCGACAACTCGTTATTAGAC	32 nt	60.6
Origami-S182	AGTGAATTGGTTATATAACTATATAAGAACGC	32 nt	56.7
Origami-S183	GTCCACTATGAAAGTATTAAGAGGAGCGGGGT	32 nt	64.4
Origami-S184	TACGAGGCCTGTCTTTCCTTATCAACCAATCA	32 nt	63.1
Origami-S185	ACAACTTTCAGCCCTCATAGTTAGAGCCCAAT	32 nt	63.1
Origami-S186	AGGAATACAAAACCAAAATAGCGAGGGGTAAT	32 nt	60.6
Origami-S187	GAGAAAACTGAAATACCGACCGTGCGGAATCA	32 nt	64.4
Origami-S188	CGACATTCCGCCTGCAACAGTGCCCGGTCAGT	32 nt	70.8
Origami-S189	TGGATAGCTTACCAGACGACGATACACATTCA	32 nt	63.1
Origami-S190	GGCAAAATCCCTTATGCAGGCGAAAATCCTGAGACGG	37 nt	68.3
Origami-S191	TTCTTACCAGTATAAAGAAAAAGCCTGTTTCTAAATTT	38 nt	58.4
Origami-S192	GCTGGTAATATCCAGACATTTTGACGCTCAATCACCAGTC	40 nt	66.8
Origami-S193	AATTAACTGAGCGCTAATATCAGAGTAAGCAGATAGCCGA	40 nt	64.7
Origami-S194	AGCCATTTGGGAATTACAGAATCAAGTTTGCCGGCATTTT	40 nt	64.7
Origami-S195	AATCCGCGGCGCAGACGGTCAATCTGACAAGAACCGGATA	40 nt	70.9
Origami-S196	TTGAGATGGTTTAATTACGAACTAACGGAACATGAGATTT	40 nt	61.6

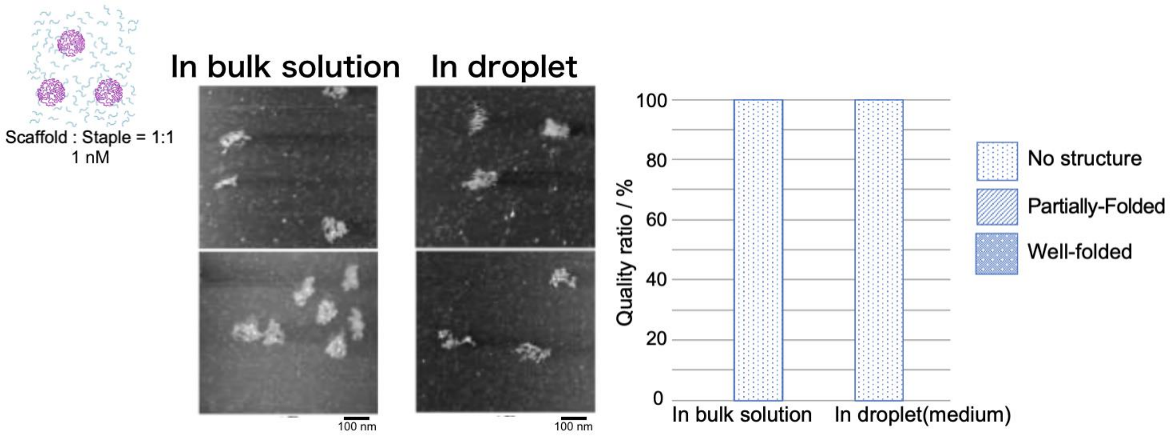

Figure S19. The “folding” efficiencies of DNA origami from a mixture of a 1:1 ratio of the scaffold to staple (1 nM). Only a non-structure state was observed in the sample in the case of DNA origami folding without starting from the purified DNA Origami sample. When the DNA solution is emulsified, the number of molecules present in the droplet varies according to the Poisson distribution. DNA origami is formed by combining approximately 200 types of staples to one scaffold. For this reason, in the droplet, it is thought that the yield of the folding was low due to the insufficient of the staple set constituting DNA origami. The yield in the bulk solution also low due to the low concentration of the DNA.
